# Unveiling a key role of oxaloacetate-glutamate interaction in regulation of respiration and ROS generation in nonsynaptic brain mitochondria using a kinetic model

**DOI:** 10.1371/journal.pone.0255164

**Published:** 2021-08-03

**Authors:** Vitaly A. Selivanov, Olga A. Zagubnaya, Yaroslav R. Nartsissov, Marta Cascante

**Affiliations:** 1 Department of Biochemistry and Molecular Biomedicine, Faculty of Biology, Universitat de Barcelona, Barcelona, Spain; 2 CIBER of Hepatic and Digestive Diseases (CIBEREHD) and Metabolomics Node at Spanish National Bioinformatics Institute (INB-ISCIII-ES- ELIXIR), Institute of Health Carlos III (ISCIII), Madrid, Spain; 3 Department of Mathematical Modeling and Statistical Analysis, Institute of Cytochemistry and Molecular Pharmacology, Moscow, Russia; Albany Medical College, UNITED STATES

## Abstract

Glutamate plays diverse roles in neuronal cells, affecting cell energetics and reactive oxygen species (ROS) generation. These roles are especially vital for neuronal cells, which deal with high amounts of glutamate as a neurotransmitter. Our analysis explored neuronal glutamate implication in cellular energy metabolism and ROS generation, using a kinetic model that simulates electron transport details in respiratory complexes, linked ROS generation and metabolic reactions. The analysis focused on the fact that glutamate attenuates complex II inhibition by oxaloacetate, stimulating the latter’s transformation into aspartate. Such a mechanism of complex II activation by glutamate could cause almost complete reduction of ubiquinone and deficiency of oxidized form (Q), which closes the main stream of electron transport and opens a way to massive ROS generating transfer in complex III from semiquinone radicals to molecular oxygen. In this way, under low workload, glutamate triggers the respiratory chain (RC) into a different steady state characterized by high ROS generation rate. The observed stepwise dependence of ROS generation on glutamate concentration experimentally validated this prediction. However, glutamate’s attenuation of oxaloacetate’s inhibition accelerates electron transport under high workload. Glutamate-oxaloacetate interaction in complex II regulation underlies the observed effects of uncouplers and inhibitors and acceleration of Ca^2+^ uptake. Thus, this theoretical analysis uncovered the previously unknown roles of oxaloacetate as a regulator of ROS generation and glutamate as a modifier of this regulation. The model predicted that this mechanism of complex II activation by glutamate might be operative *in situ* and responsible for excitotoxicity. Spatial-time gradients of synthesized hydrogen peroxide concentration, calculated in the reaction-diffusion model with convection under a non-uniform local approximation of nervous tissue, have shown that overproduction of H_2_O_2_ in a cell causes excess of its level in neighbor cells.

## Introduction

Glutamate is the most abundant excitatory neurotransmitter in the central nervous system (CNS) [1]. The glutamatergic neurotransmission plays a crucial role in synaptic plasticity, which is in charge of cognition, memory, and learning [2]. It is also highly required in synaptic induction and elimination, cell migration, differentiation, and death [3].

Since glutamate permeation through the blood-brain barrier is highly restricted [4], the cells should synthesize it endogenously. Most of the brain glutamate is *de novo* synthesized from Krebs cycle intermediate α-ketoglutarate [5], by aminotransferase reactions. However, a novel intra-neuronal metabolic pathway converting urocanic acid to glutamate after UV-exposure is also reported [6].

Ambient extracellular glutamate concentration should be kept below 0.5–5 μM [7] to prevent excessive glutamate receptor stimulation. Glutamatergic synapses assemble almost one-third of all excitatory synapses in CNS. Glutamate can induce neuronal dysfunction and degeneration when present in abnormally high extra-cellular concentrations [8]. Since late sixties of the past century, this process is referred to as glutamate excitotoxicity when John W. Olney extended the ability of parenterally administered glutamate to kill neurons in the hypothalamus and hippocampus [9]. Glutamate is the major excitatory neurotransmitter in the brain, and its excessive release leads to repeated depolarization-repolarization cycles in glutamate terminals. Consequently, the degeneration of postsynaptic neurons occurs due to the increase in calcium influx, mainly through N-methyl-D-aspartate (NMDA) ionotropic receptor activation [10]. There is a wide-observed pathway of cell death in the brain induced by glutamate excess for various pathological processes such as stroke/ischemia, temporal lobe epilepsy, Alzheimer’s disease, and amyotrophic lateral sclerosis [11–13]. Neuroinflammation can be considered as the process which has a key role to prompt excitotoxicity. In particular, inflammation causes tryptophan catabolic transformation into an agonist of NMDA receptors, probably increasing glutamate concentrations in the brain interstitial fluids (ISF) [14]. Tumor necrosis factor alpha (TNF-α) can potentiate glutamate-mediated cytotoxicity by two complementary mechanisms: indirectly, by inhibiting glutamate transport on astrocytes, and directly, by increasing the localization of ionotropic glutamate receptors to synapses [15].

Recent advances in brain energy metabolism studies strongly suggest that glutamate receptor-mediated neurotransmission is coupled with molecular signals that switch-on glucose utilization pathways to meet neurons’ high energetic requirements [16]. Failure to adequately coordinate energy supply for neurotransmission ultimately results in a positive amplifying loop of receptor over-activation leading to neuronal death. While the neurotransmitter’s homeostatic balance is disrupted, elevated glutamate levels in the extracellular environment of the central nervous system play a pivotal role in neurodegeneration in acute CNS injuries [17]. Glutamate-induced excitotoxicity is mainly linked to an impaired ability of glial cells to reuptake and respond to glutamate. This impairment is considered a common hallmark in many neurodegenerative diseases, including Parkinson’s disease [18] and tumor-associated epilepsy [19]. Although glutamate‐dependent excitotoxicity is the primary mechanism in neuronal apoptosis, the rapid excitation of neurons due to a massive influx of calcium without the neurotransmitter glutamate level increase (glutamate‐independent excitotoxicity) is also a contributing factor, especially in traumatic brain injury [20].

Our study aimed at a deep qualitative analysis of the destructive intracellular processes, initiated by excessive accumulation of glutamate, that can lead to cell death. Therefore, we consider glutamate transport into the neuronal cells and its interactions with cellular metabolism that can promote harmful effects. Five types of glutamate transporters, called Excitatory Amino Acid Transporters (EAAT) in the general classification, on the cytoplasmic membrane have been caracteryzed. All EAATs belong to a Solute carrier 1 family. The types of EAATs differ by their affinity to substrate and capacity to substrate translocation [21], but share the same mechanism of transport, which is accounted for in our analysis (see section Models). EAATs protomers also possess an anion channel activity to avoid excessive membrane depolarization. This anion activity is associated but not stoichiometrically coupled to substrate translocation [22].

EAATs are secondary active transporters with general stoichiometry. The membrane glutamate transport process is electrogenic and facilitated by negative transmembrane potential, as a net of two positive charges is translocated inside the cell during each transport cycle [23]. In one catalytic cycle, the protein binds and moves into the neuron one molecule of glutamate, three sodium ions, and one proton, and carries out one potassium ion. The sequence of elementary events of the protein transport cycle is conserved among EAATs types. The protonated transporter form binds a glutamate molecule after the first sodium ion binding [24]. Thus, glutamate is the only substance that flows up counter to its concentration gradient, as the other co-transported ions flow down corresponding concentration gradients. Sodium ions are essential for glutamate binding, and potassium ion is necessary for transformation back to the initial state. Anion activity of EAATs, associated with the transport cycle, is glutamate and sodium–gated [25]. Our theoretical study accounts for this mechanism of glutamate transport.

The glutamate molecules are stored in presynaptic vesicles, which contain up to 100 mМ of glutamate [5] concentrated by vesicular glutamate transporters using proton gradient generated by V-type H+-ATPase across synaptic vesicular membrane [26].

**Fig 1:**
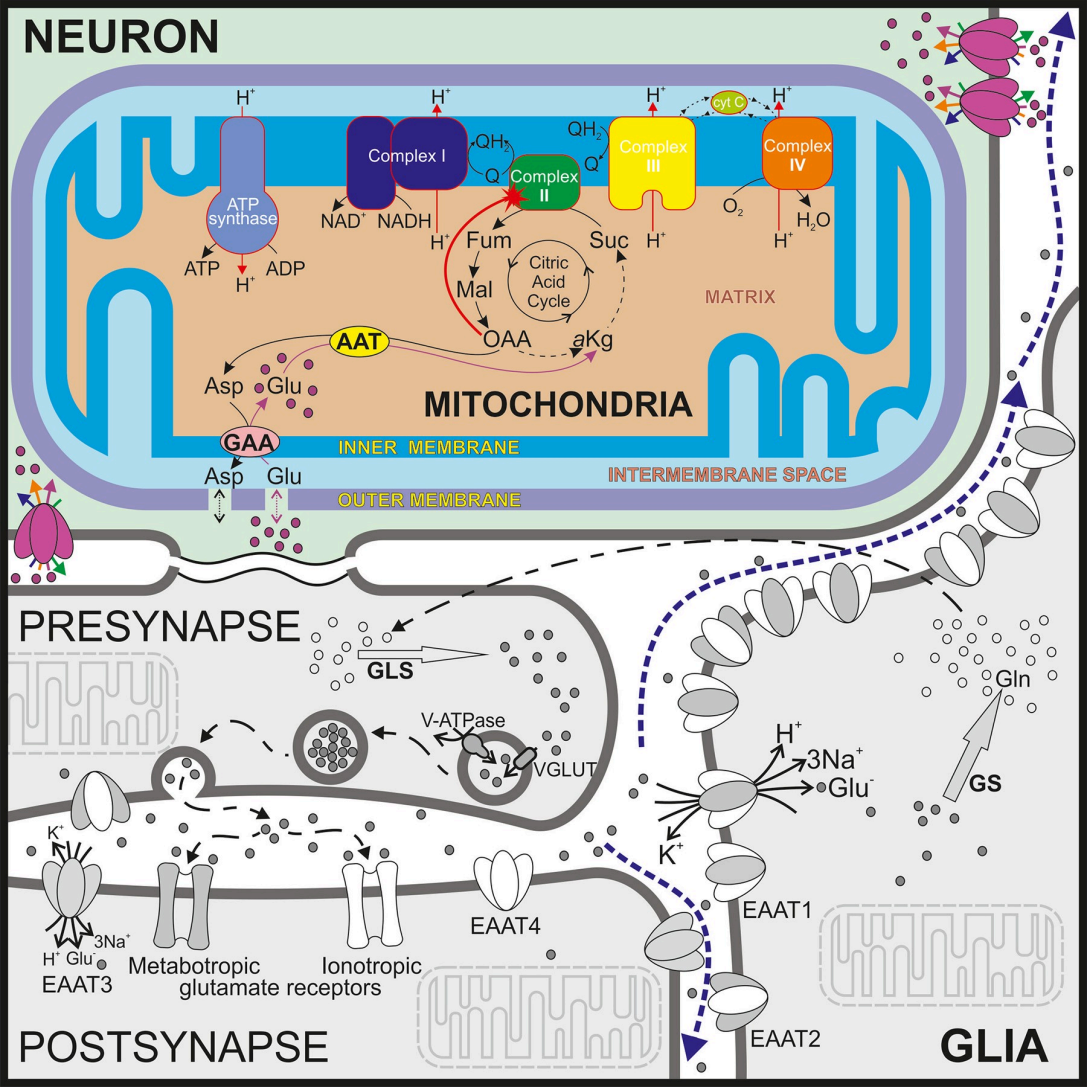
Extended scheme of glutamate involvement in complex II regulation. While a whole system of principal glutamate reactions is shown, the subject of the model is painted in color. It illustrates the implementation in the model mechanism of complex II activation by glutamate. The processes not included directly in the model are shown in grey. The most common glutamate-involved processes are separated in the different cells. Several types of transporters of the EAAT family are indicated in appropriate membranes. The metabolic reactions of GLS, GS, GDH, and AAT are shown in local areas of a neuron (presynapse and postsynapse) and glia. Dashed blue arrows indicate spillover of glutamate. According to the specificity of glutamate transporter localization [3, 21, 23], EAAT3 is considered as the carrier used in the model. The reactions in a neuroplasm (for example glycolysis, NADH metabolism, etc) are omitted for the clearaty of presentation.

Glutamatergic neurotransmission begins with calcium-dependent glutamate efflux from the nerve terminals to the synaptic cleft after cytoplasmic membrane depolarization. Then the neurotransmitter interacts with metabotropic and ionotropic receptors on the postsynaptic neuronal terminal membrane. These receptors also have ion channel activity. The activation leads to the influx of calcium and sodium ions and potassium efflux from the postsynaptic nerve terminals and depolarization of the corresponding neuron membrane. Such a depolarization terminates after clearance of synaptic cleft from glutamate after action potential generation [23]. That part of glutamate, which was not removed from the synaptic cleft by glutamate transporters, can diffuse, filling the whole extracellular space. This process, called spillover, which is crucial in intersynaptic crosstalk and so in synaptic plasticity [1, 2], is considered in our analysis as the primary source of extracellular glutamate affecting cellular metabolism (Fig 1).

Glial cells enclosing glutamatergic synapse remove up to 90% of released glutamate, which is rapidly converted into glutamine by glutamine synthetase (GS). A considerable proportion of glutamate undergoes oxidative degradation in astrocytes, where oxidation of glutamate is initiated by transamination catalyzed by an aminotransferase (AAT), or oxidative deamination catalyzed by glutamate dehydrogenase (GDH) [27]. Synthesized glutamine is released into extracellular space and uptaken by neurons, where glutaminase (GLS) converts it back to glutamate [28]. The neuronal glutamate is also converted to GABA, the primary inhibitory transmitter in the CNS. Moreover, glutathione is synthesized including glutamate as a composing compound. It is remarkable, that glutamate not only contributes to brain energy buffering through the transformation to α-ketoglutarate by GDH or AAT [5]. Together with direct metabolic reactions supplying ATP synthesis, glutamate can affect cellular energy metabolism as a potent regulator. We previously found that glutamate activates H_2_O_2_ synthesis in nonsynaptic brain mitochondria [29]. Specifically:

- the activation was observed in the presence of rotenone (inhibitor of respiratory complex I) and high concentrations (5 mM) of succinate as a substrate for the respiratory chain (RC);
- the concentration dependence of the activation appears as a stepwise process, so that low glutamate concentrations <100 μM induce maximal activation and further increase of glutamate does not further activates H_2_O_2_ production;
- low glutamate concentration accelerates uptake of Ca^2+^;
- glutamate does not further increase the antimycin-induced activation of H_2_O_2_ generation and vice versa;
- uncouplers of electron transport and ATP synthesis prevent the activation of H_2_O_2_ generation.

These experimental data indicate that glutamate affects the RC operation. Respiratory complex II is the main gate for electron influx into the RC under the considered experimental conditions (excess of succinate in the presence of rotenone). The listed above effects of glutamate, at first glance, seem to be consistent with a hypothesis that glutamate activates complex II. Panov et al. proposed that glutamate can attenuate complex II inhibition by oxaloacetate [30]. This hypothesis is based on the well-known facts that oxaloacetate is an inhibitor of complex II [31, 32]. Two processes contribute directly to the attenuation of oxaloacetate inhibition. First, aspartate transaminase transforms oxaloacetate into aspartate, taking an amino group from glutamate and transforming it into alpha-ketoglutarate. Second, aspartate is forced out of mitochondria in exchange for external glutamate. In this way, glutamate causes a decrease of oxaloacetate concentration. Further, this process is referred to as activation of complex II by glutamate.

Complex II inhibition by oxaloacetate is especially notable in brain cells [33]. Therefore, the interaction of glutamate with this process can play an essential regulatory role in the brain. The scheme of respective reactions is shown in Fig 1. When succinate is added as a substrate for the RC, its subsequent oxidation produces fumarate, malate, and then oxaloacetate. Oxidation of the latter in the Krebs cycle requires acetyl Co-A, a product of pyruvate dehydrogenase reaction. If pyruvate supply is not sufficient, oxaloacetate concentration can increase and inhibit succinate oxidation in complex II [32].

**Fig 2:**
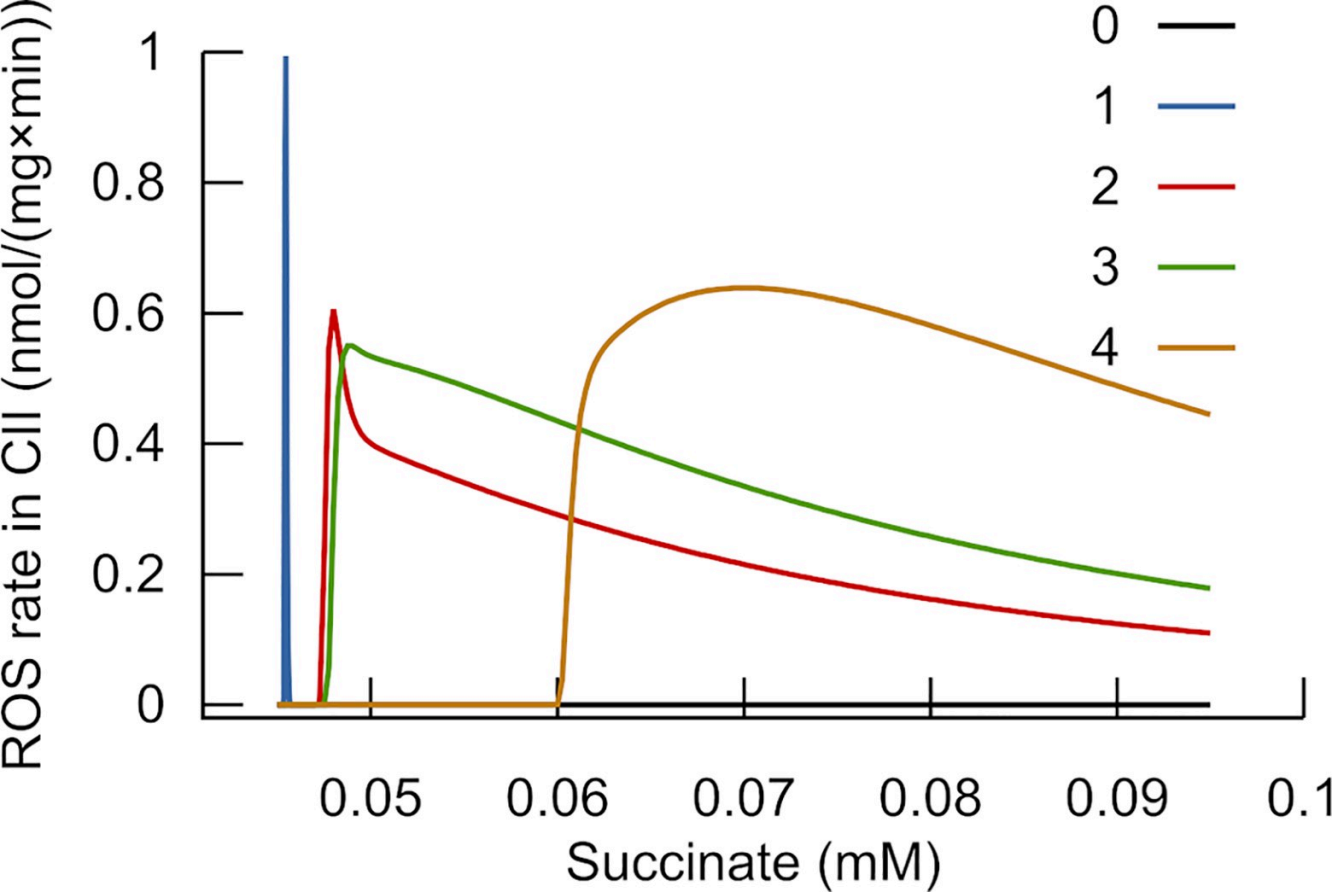
Simulated dependence of ROS production in functionally isolated complex II on succinate concentration. The model simulated ROS generation in complex II functionally isolated by rotenone (setting to 0 the binding constant of quinone to complex I) and myxothiazol (changing the binding constant of QH_2_ to the Qo site of complex III). Curve “0” simulates absence of myxothiazol (binding QH_2_ to the Qo site not changed, 700 (nmol/mg)^-1^s^-1^), curves 1–4 characterize various extents of inhibition by myxothiazol. The remaining activity is reflected by the changed binding constant (relative to the unchanged one) 4×10^−8^ for curve 1, 1.4×10^−6^ for curve 2, 2×10^−6^ for curve 3, 4×10^−6^ for curve 4. The values of ROS generation are normalized to the maximal peak (obtained for curve 1).

We previously developed a detailed kinetic model of the RC functioning [34–36]. We used software Mitodyn [37] that implements an extended version of this model to analyze the observed effects of glutamate in depth. To our knowledge, no mathematical models were developed previously to analyze the consequences of complex II inhibition by oxaloacetate and the interaction of glutamate with this process. The **first objective** of this contribution was to check whether the hypothetical mechanism of complex II activation by glutamate underlies the glutamate effects on the RC observed in [29]. To this end, we simulated data of [29] with a model that implements the above described hypothesis of the RC activation by glutamate. Specifically, we qualitatively simulated the following data reported in [29]: the stepwise activation of ROS generation summarized in Fig 2F, Ca^2+^ uptake presented in Fig 4A, combined effect of inhibitors myxothiazol and antimycin shown in Fig 5C and an uncoupler in Fig 6 of [29]. Suppose glutamate, indeed, provokes such significant changes in ROS production, and these changes are relevant in situ. In that case, regulation of the RC operation by glutamate in neuronal cells may have significant physiological consequences. Revealing the possible outcome of glutamate accumulation in neuronal cells *in situ* under excessive stimulation was the **second objective** of this contribution.

We qualitatively simulated the data of Lobysheva et al. [29], to get a more in-depth insight into the mechanism of glutamate interaction with the RC and ROS production in complexes II and III. The model used for the simulations assumed the mechanism of glutamate interaction with mitochondrial metabolism, as Fig 1 shows. After validating this hypothesis by the experimental data simulation, we predicted the possible effects of glutamate in neuronal cells *in situ*, including the visualization of ROS propagation dynamics to the neighbor cells, using a three-dimensional reaction-diffusion model.

## Results

### I. Isolated mitochondria

#### 1. ROS generation by functionally isolated complex II

ROS production in complex II, functionally isolated from complexes I and III by applying inhibitors rotenone and myxothiazol, was studied experimentally [38–40]. These studies revealed a bell-shaped dependence on succinate at low concentrations. Our simulation of ROS production in the flavin site of complex II (according to Messner and Imlay [41]) with inhibited electron transport through complexes I and III reproduced such a bell-shaped peak at low succinate concentrations (~50 μM). The simulation shows a much steeper drop in ROS generation than observed in the experiments (curve 1 in Fig 2) if myxothiazol completely inhibits upstream electron flow. However, if inhibition by myxothiazol is not complete, but a small part of complexes III remains active, the drop in ROS generation by complex II is not so steep (curves 1–4 in Fig 2). It becomes similar to that observed in the experiments. According to the model, this dependence is bell-shaped because the fraction of flavin semiquinone reaches its highest value at low succinate concentration. With succinate increase, flavin becomes fully reduced, and the concentration of ROS generating semiquinone decreases.

#### 2. Stepwise activation of ROS production by glutamate

**Fig 3:**
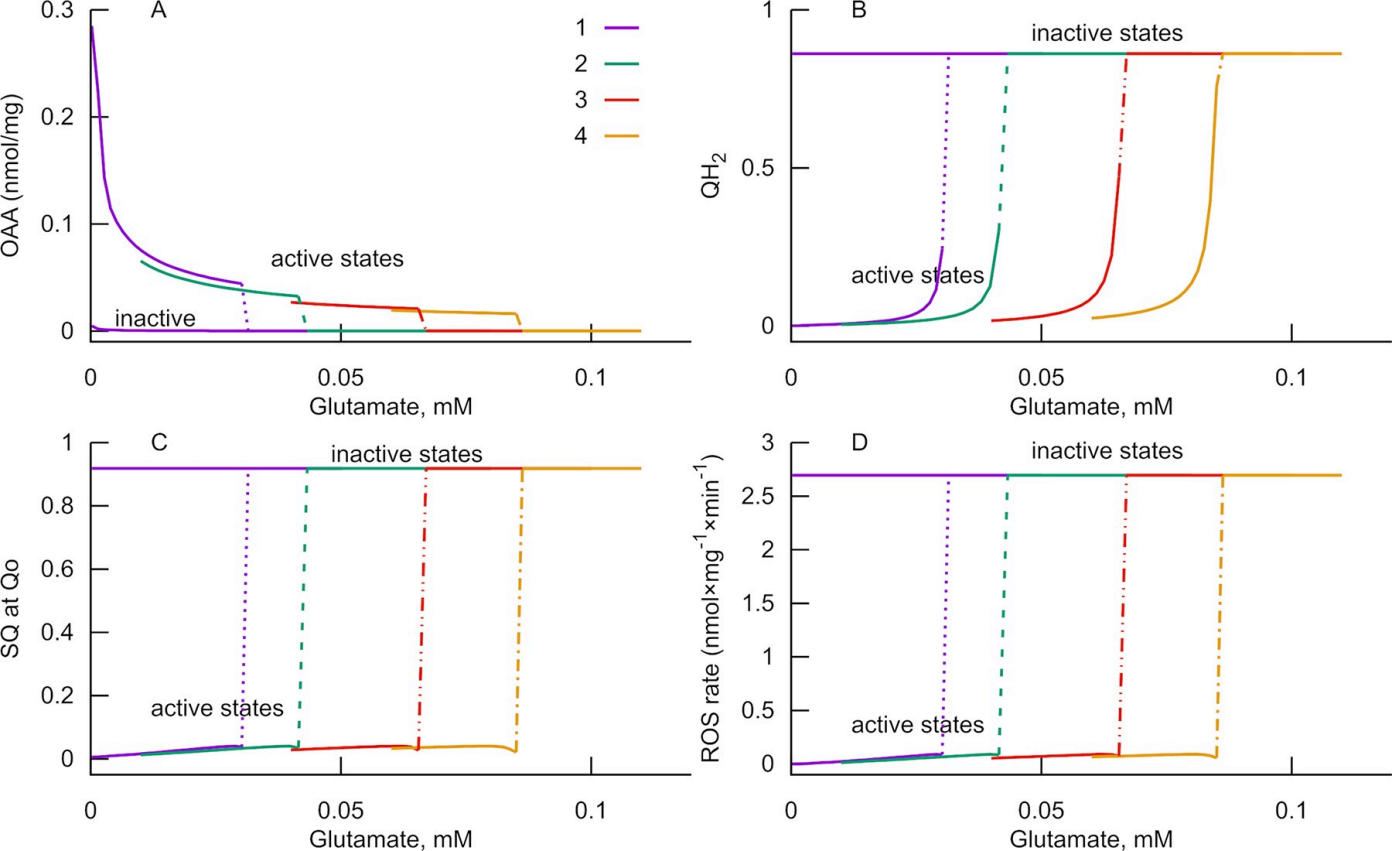
Stepwise activation of ROS generation by glutamate. Gradual increase of glutamate concentration leads to abrupt changes (bifurcation) in the RC state (indicated by dotted and dashed lines). Mitochondrial oxaloacetate concentration decreases (A). Free ubiquinone becomes practically completely reduced (B, QH_2_ ratio to the total ubiquinone amount is shown). The fraction of semiquinone (SQ) formed at the Qo site of complex III increases (C). ROS generation in complexes II and III increases (D, the ratio of ROS accumulated during the first minute to the amount of these complexes is shown). The maximal rate of ROS generation corresponds to that obtained experimentally when the rate constant of ROS formation is 2×10^−5^ s^-1^. Curves 1–4 are obtained with different values of rate constant of FAD reduction by succinate in the flavin center of complex II (in (nmol/mg)^-1^s^-1^), 1: 175, 2: 131, 3: 88, 4: 70.

It was found [29] that glutamate, applied in low concentrations, induces a stepwise increase of mitochondrial ROS production. Fig 3 shows a simulated abrupt change in the RC state related to ROS generation as a function of glutamate concentrations.

This discontinuous change is a normal consequence of commonly recognized reactions taking place in respiratory complexes and mitochondrial matrix, implemented in the differential equations of the model. The model implements an aminotransferase reaction that, using glutamate as a substrate, transforms oxaloacetate into aspartate. This reaction decreases oxaloacetate concentration (Fig 3A) and, respectively, complex II inhibition. Such activation of complex II at moderate workloads results in the reduction of free ubiquinone pool (Fig 3B) and, consequently, in the deficiency of oxidized form (Q). Since the latter is the substrate acceptor of electrons in Qi site of complex III, its deficiency causes the electron, accepted from QH_2_ at Qo site and designed for Q, to be accepted by molecular oxygen, giving superoxide radical O2-. Thus, the switch of main electron flow between the two acceptors: Q or O_2_ in complex III underlies the switch between the two branches of steady states. In this way, when it reaches a critical concentration corresponding to the bifurcation point, glutamate stimulates positive feedback: activation of complex II leads to Q deficiency. The latter slows down QH_2_ oxidation, resulting in higher ubiquinone reduction that aggravates Q deficiency. This positive feedback leads to the switch to a different branch of steady states where the level of semiquinone radicals (SQ) at Qo site of complex III is high (Fig 3C), and, respectively, the rate of ROS generation (Fig 3D) is high. The maximal levels of ROS generation shown in Fig 3D quantitatively correspond to those measured experimentally in [29] when the rate constant of ROS generation is 2×10^−5^ s^-1^. However, we realize that it could be higher because the model does not account for ROS inactivation in the real object. This change in complex III functioning leads to a temporal increase of flavin semiquinone radicals in complex II. Thus, the reason for an abrupt switch in ROS generation is the multistationarity of the RC operation [34–36].

Practically the two branches of steady states were calculated as follows. Starting from a point corresponding to an "active" steady state the program solves many initial value problems (IVP), applying a small increment in glutamate concentration, thus calculating steady states corresponding to the incremented parameter. The curve of active steady states is calculated by repeating this operation, taking each obtained solution as an initial point for a subsequent calculation. At some iteration, it reaches a bifurcation point where a solution discontinuously changes to a branch indicated in Fig 3 as "inactive". This switch is shown in Fig 3 by dashed lines. After further incrementing and subsequent decrementing the glutamate concentration, the solution stays in this "inactive" branch, even after decreasing glutamate concentration below the bifurcation point. In this way, the program finds two branches of stable steady states existing in the same interval of parameters. The system reaches one or another of them depending on the initial values of variables. Probably there exist a branch of unstable steady states between them and connected to the "active" branch at the bifurcation point. However, the applied method does not allow to locate unstable steady states, and the "active" branch seems terminated at the bifurcation point, whereas "inactive" states exist for all possible glutamate concentrations. Probably, the switch to this branch corresponds to excitotoxicity and leads to cell death. It is possible that perturbation of other model parameters can induce a switch back to an "active" state, but it is a subject of future investigation.

The comparable glutamate dependence of ROS generation shown in Fig 2 of [29] also reaches saturation at low glutamate concentration. Still, it is not as steep as the curves presented in Fig 3D. However, a variation of the rate constant of succinate binding to complex II leads to a shift of the bifurcation point. Therefore, we believe that the switch between the steady states described in the model, in principle, corresponds to the stepwise dependence observed in the experiment. The latter is not so steep as in the model because of the RC characteristics heterogeneity in the mitochondrial suspension.

#### 3. The effect of the RC uncoupling on ROS production

**Fig 4:**
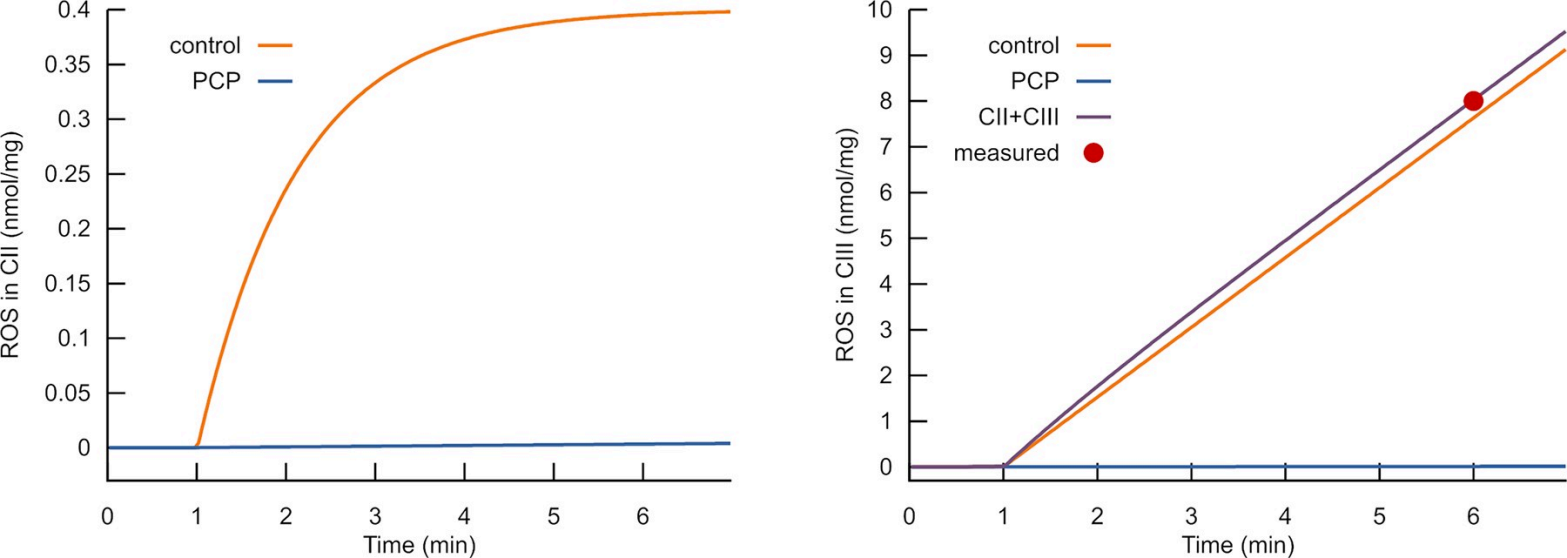
Uncoupling prevents the glutamate stimulated ROS production. Calculated ROS accumulation produced in complexes II (A) and III (B) after complex II activation by glutamate in the presence (blue curves) and absence (orange curves) of an uncoupling agent. Uncoupling is simulated as an increase of proton leak rate constant (from 2 to 40 s^-1^×mV^-1^). The calculations stimulated the application of uncoupler PCP, as reported in Fig 6 of [29].

Fig 4 shows an impact of uncoupler simulated as an increase of proton leak. Glutamate increases ROS production in complexes II and III by the mechanism outlined above. Experimentally, uncouplers, such as PCP, prevent an increase of ROS production stimulated by glutamate. The model simulates this effect of uncouplers. According to the model, ROS production decreases because ubiquinol (QH_2_) produced by the activated complex II is quickly oxidized for proton translocation giving Q, which is necessary to avoid the switch to excessive ROS production. Thus, the increased demand for active electron and proton transport in the respiratory chain, as an effect of uncouplers, causes a decrease in ROS production. In this case, activation of complex II by glutamate does not result in increased ROS production.

Although both complexes respond similarly to glutamate stimulation without an uncoupler, the time courses of their responses are different. Whereas in complex III the rate is constant, in complex II it is high at the beginning, but then it rapidly decreases. The latter dynamics reflect a fast increase of flavin semiquinone radical content immediately following the switch to the new steady state. Then flavin became fully reduced, and the ROS production rate drops down.

#### 4. Application of antimycin and myxothiazol together with glutamate

**Fig 5:**
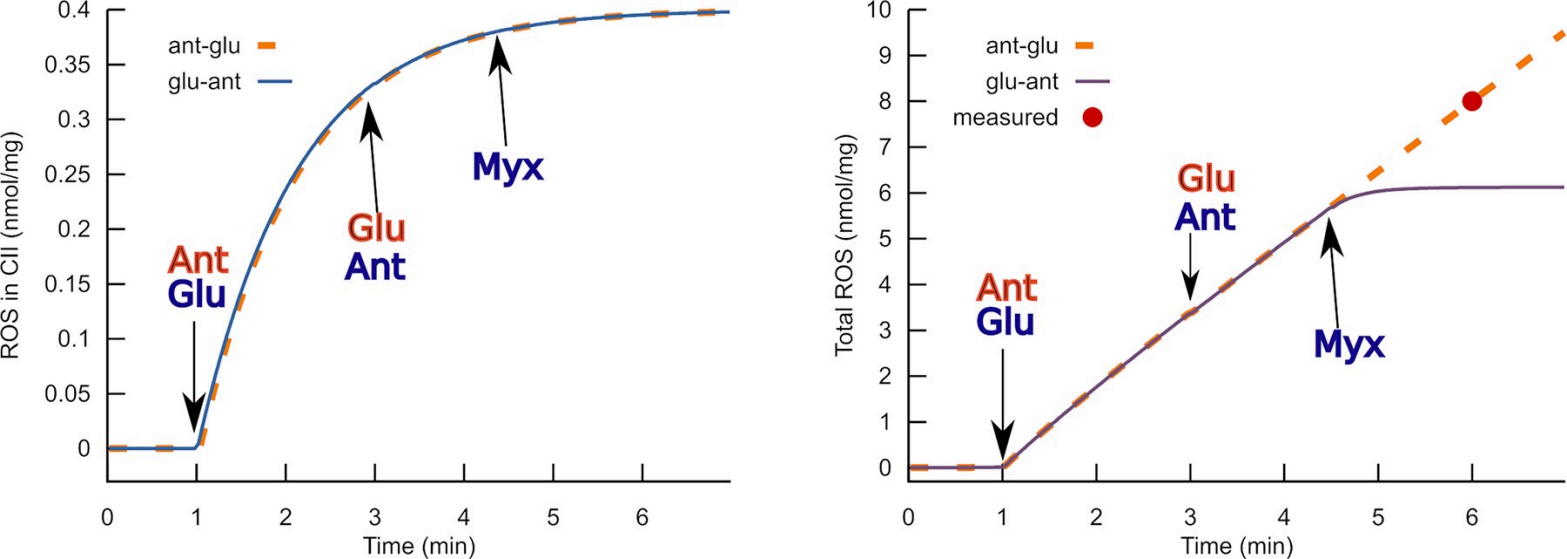
Simulated ROS production response of complexes II (A) and III (B) to combinations of antimycin, glutamate, and myxothiazol. The action of antimycin simulated as a block of ubiquinone binding and dissociation in the N-center of complex III (rate constants of these reactions are 0). Myxothiazol blocks ubiquinol binding in the Qo-center of complex III (corresponding rate constant is 0). Glutamate activates complex II as described in the Introduction. The orange and blue curves simulate different orders of additions. Orange: glutamate is added before antimycin, blue: antimycin is added before glutamate. These simulations qualitatively reproduce a direct record of H_2_O_2_ accumulation varying the order of addition of glutamate and antimycin, as shown in Fig 5 of [29]. The code, initial values and rate constants corresponding to this simulation is available at https://github.com/seliv55/cell_mito/tree/fig6.

Fig 5 shows that glutamate, as well as antimycin, induces an increase in ROS production. The subsequent application of the second of these two active compounds does not further accelerate ROS production. The absence of additivity in the effects of these stimulators of ROS productions supports the idea of bistability in the RC operation. Any of these compounds switch the RC into the steady-state, where ROS production is maximally stimulated. The subsequent addition of the other compound does not change this steady-state.

Although the effects of glutamate and antimycin are similar, the delays between the effector addition and the response are different. Complex III practically immediately responds to antimycin and delays in response to glutamate. This difference is understandable: antimycin affects complex III directly, blocking Q reduction in the Qi site, whereas glutamate affects complex III after reducing the ubiquinone pool. In complex II, the situation is different. The content of flavin semiquinone radicals rapidly increases when the ubiquinone pool is highly reduced. This threshold of ubiquinone reduction is reached faster when glutamate stimulates complex II. When ubiquinone became practically completely reduced, the content of flavin semiquinone radicals decreases because flavin became completely reduced. Then the ROS production rate in complex II drops.

According to the model, myxothiazol itself induces ROS production in complex II similar to that caused by antimycin or glutamate. It agrees with [42], where stimulation of ROS production by mitochondria respiring on succinate in the presence of myxothiazol was found. The delay for the complex II response is a bit shorter than that for antimycin because myxothiazol stops the reaction of QH_2_ oxidation in complex III. In contrast, antimycin accelerates the reaction of ROS production that oxidizes QH_2_. This oxidation delays the complete QH_2_ reduction that is translated into an increase in flavin semiquinone content.

#### 5. Acceleration of Ca^2+^ uptake by mitochondria

**Fig 6:**
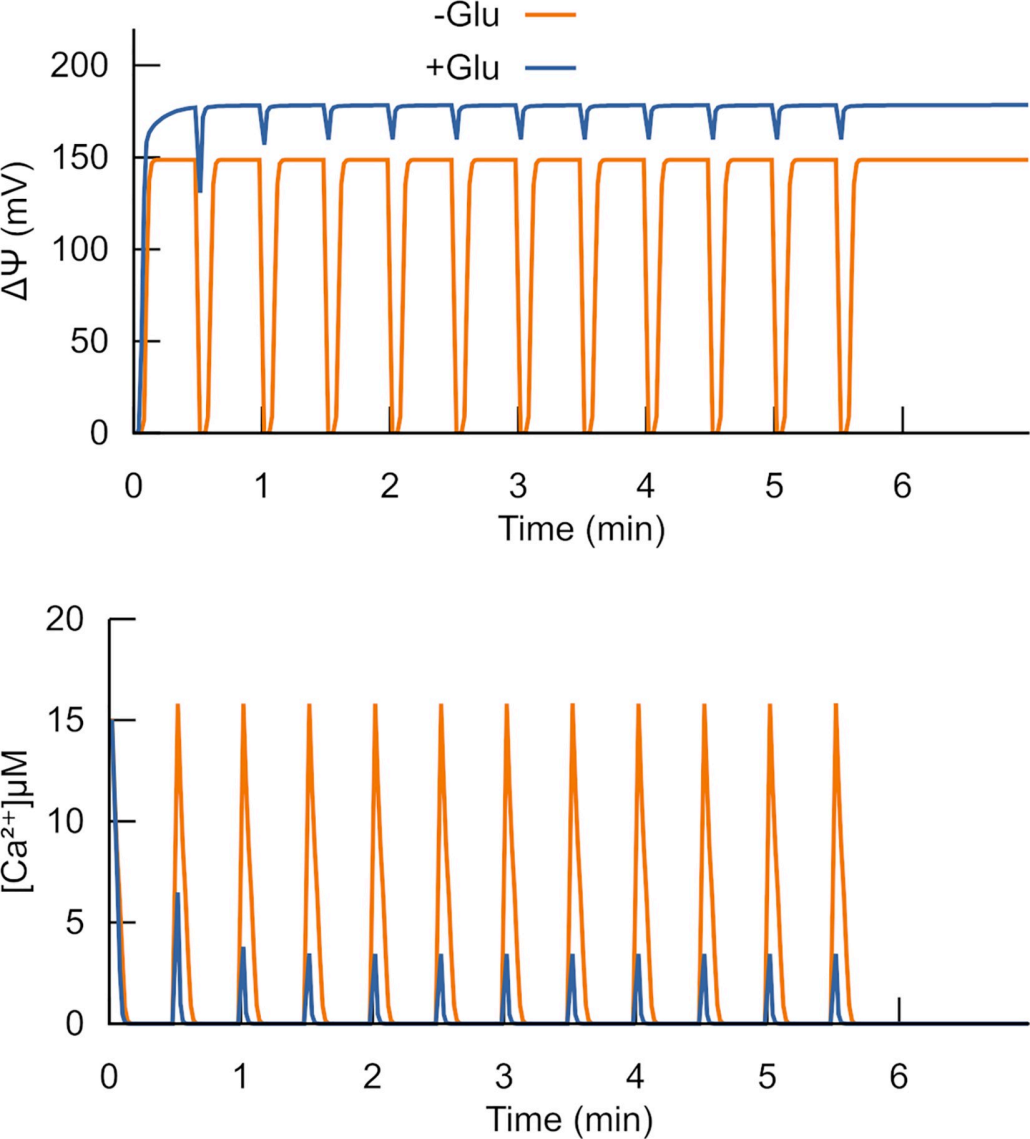
Glutamate—activated Ca^2+^ uptake by mitochondria. The calculated dynamics of transmembrane potential (A) and Ca^2+^ (B) in response to multiple additions of 20 nmol Ca^2+^. The uptake of positive Ca^2+^ ions discharges the membrane. After the uptake, Δψ recovers. The model accounts for the uptake as JCa = P·Δψ·([Ca^2+^]o-[Ca^2+^]i)· exp(zFΔψ/RT) /(1-exp((zFΔψ/RT)), here JCa is the uptake flux of Ca^2+^, P = 0.005 s^-1^×mV^-1^ is permeability coefficient, Δψ is the transmembrane potential, z = 2 is the ion charge, R is the ideal gas constant, T is the temperature in kelvins, F is Faraday’s constant. RT/F is ~25.693 mV. d(Δψ)/dt = F/C · 2·JCa describes the JCa dependent change of Δψ. C is the electric capacity of the membrane, F/C = 500 mV·nmol^-1^·mg protein. The simulations correspond to the direct record of Ca^2+^ uptake reported in Fig 4 of [29].

Ca^2+^ enters mitochondria through Ca^2+^ uniporter forced by the transmembrane electric potential (Δψ). According to the simulations shown in Fig 6, glutamate accelerates Ca^2+^ entry. This acceleration of Ca^2+^ entry is in accordance with the measurements reported in Fig 4 of [29].

Complex II stimulation by glutamate is the reason for faster Δψ recovery. The entry of positively charged Ca^2+^ ions decreases the Δψ. In this way the effect of Ca^2+^ is similar to that of uncouplers (see part 3 of Results). As in the case of uncouplers, glutamate does not induce the switch to ROS production but stimulates effective electron transport that recovers Δψ faster. The faster Δψ recovery causes more rapid Ca^2+^ uptake.

It should be noted that Ca^2+^ uptake was simulated to show one more phenomenon that supports the suggested here mechanism of complex II activation by glutamate. We realize that Ca^2+^ metabolism is complex, it includes Ca^2+^ / H^+^ antiport, Ca^2+^ / Na^+^ exchange, binding by proteins, precipitation as phosphate salt [43, 44]. However, details of this metabolism are out of the scope of the presented study. Our objective was to demonstrate that activation of the RC by glutamate provides faster uptake of externally added Ca^2+^, higher levels and faster recovery of Δψ. Since the Δψ-dependent Ca^2+^ uptake of added Ca^2+^ is the main way of its transport under the considered conditions, only this way was implemented in the simulations shown in Fig 6. Since inside mitochondria, Ca^2+^ is mainly bound to proteins or precipitated, its concentration inside was constant in the model. Δψ here is calculated as described in equations H.1-H.7 in [35], taking into account that complex I is inhibited by rotenone and Δψ is maintained by electron transport in complex III.

### II. Intact cells in situ

**Fig 7:**
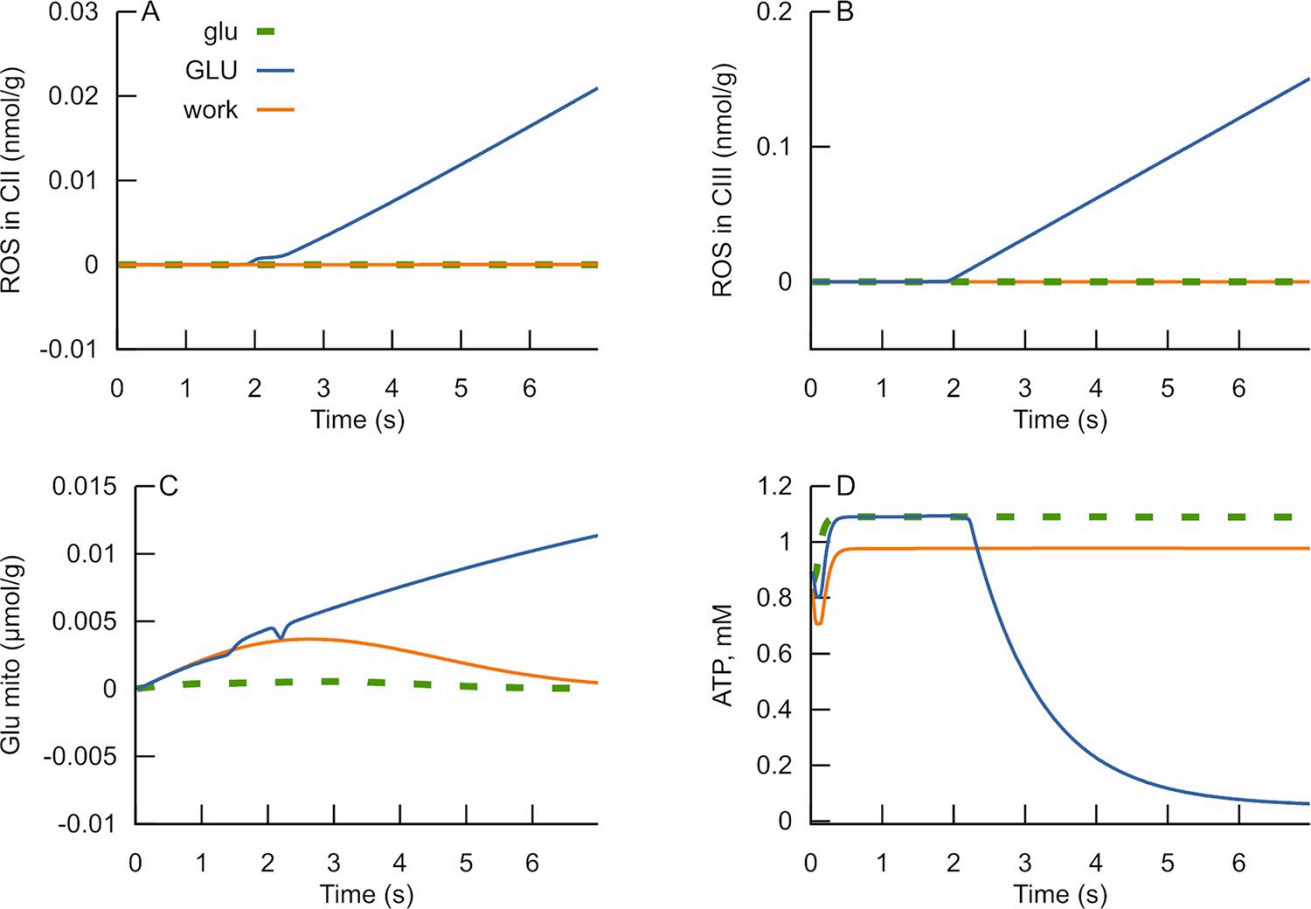
Cellular ATP levels and ROS production at various glutamate concentrations and workload (ATPase). Dynamics of ROS production rate in complex II (A), complex III (B), mitochondrial glutamate (C), and cellular ATP levels (D) at various rates of glutamate uptake and cellular ATPase activity. Dashed green curves in panels A-D, labeled as “glu,” represent simulations of conditions when excitation results in uptake of 1 μmol of glutamate per 1 g of tissue and ATPase rate of 1nmol×g^-1^×s^-1^. Blue curves in panels A-D marked as “GLU” represent simulations of conditions when excitation results in glutamate uptake of 3 μmol/g of tissue and the sameATPase rate. Orange curves labeled as “work” represent simulations of conditions when excitation results in glutamate uptake of 3 μmol/g of tissue and ATPase rate of 10nmol×g^-1^×s^-1^. The code, initial values and rate constants corresponding to this simulation are available in https://github.com/seliv55/cell_mito/tree/fig8.

Astrocytes, as widely accepted, mediate the bulk of glutamate uptake in the brain. Petr et al. showed that the biochemical uptake assays could dramatically overemphasize neuronal uptake [45]. Therefore, glutamate dynamics and glutamate transporter function should be studied *in situ* [46–48]. Such studies have shown that synaptic activity generates extrasynaptic glutamate dynamics with magnitude and duration sufficient to activate extrasynaptic glutamate receptors. Okubo et al. measured extracellular glutamate dynamics in response to a series of stimulating pulses at 100 Hz using optical glutamate sensors [49]. They found that average extracellular glutamate concentration after fife stimulating pulses at 100 Hz reached 20μM. This glutamate was uptaken during ~15 ms after the fifth pulse. Pinky et al. [46] found that after more prolonged stimulation (50–100 pulses), the extracellular glutamate reached a higher concentration, above the threshold for optical sensor saturation. This increased glutamate concentration in the extracellular space persists during some seconds after the stimulation [46]. If the extracellular volume is close to that for the intracellular one, then extracellular 20 μM corresponds to 10 nmol/mL of tissue. If this amount is uptaken during 15 ms and the rate remains the same during and after prolonged stimulation, then after 5 s of stimulation, more than 3 μmol/mL of tissue can be uptaken by nervous cells. A simulation of the possible consequences of this uptake is shown in Fig 7.

The model used for such simulations is an extension of the model used to analyze glutamate effects on isolated mitochondria described above. This extension includes a mathematical description of glutamate transport into the cells from extracellular space and a summation of glutamate released from neighboring synapses into extracellular space, cytosolic reactions of malate—aspartate shuttle, *Na⁺*/*K⁺* ATPase, integral cellular ATPase, ATP synthase, glycolysis in simplified form, NADH metabolism (see Methods).

Fig 7 simulates the dynamics of ROS production rate, mitochondrial glutamate level, and ATP concentration in neuronal cells in situ. At low glutamate concentrations in mitochondria and slow ATP consumption, cells maintain a high level of ATP (dashed curves in panels A-D, labeled as “glu”). The increase of glutamate concentration resulted in the RC switch to an inactive state with high ROS generation rate and decreased ATP synthesis rate. In this situation, ATP levels drop (blue curves in panels A-D, labeled as “GLU”). Suppose under the latter conditions of glutamate uptake, ATPase activity increases an order of magnitude. In that case, mitochondrial glutamate concentration does not increase because the entered glutamate is transformed into α-ketoglutarate and consumed in the Krebs cycle. Thus, the increased ATPase activity prevents a rise in ROS production, and high ATP levels are maintained.

### III. Spatial-time gradients of hydroperoxide in a local brain area

**Fig 8:**
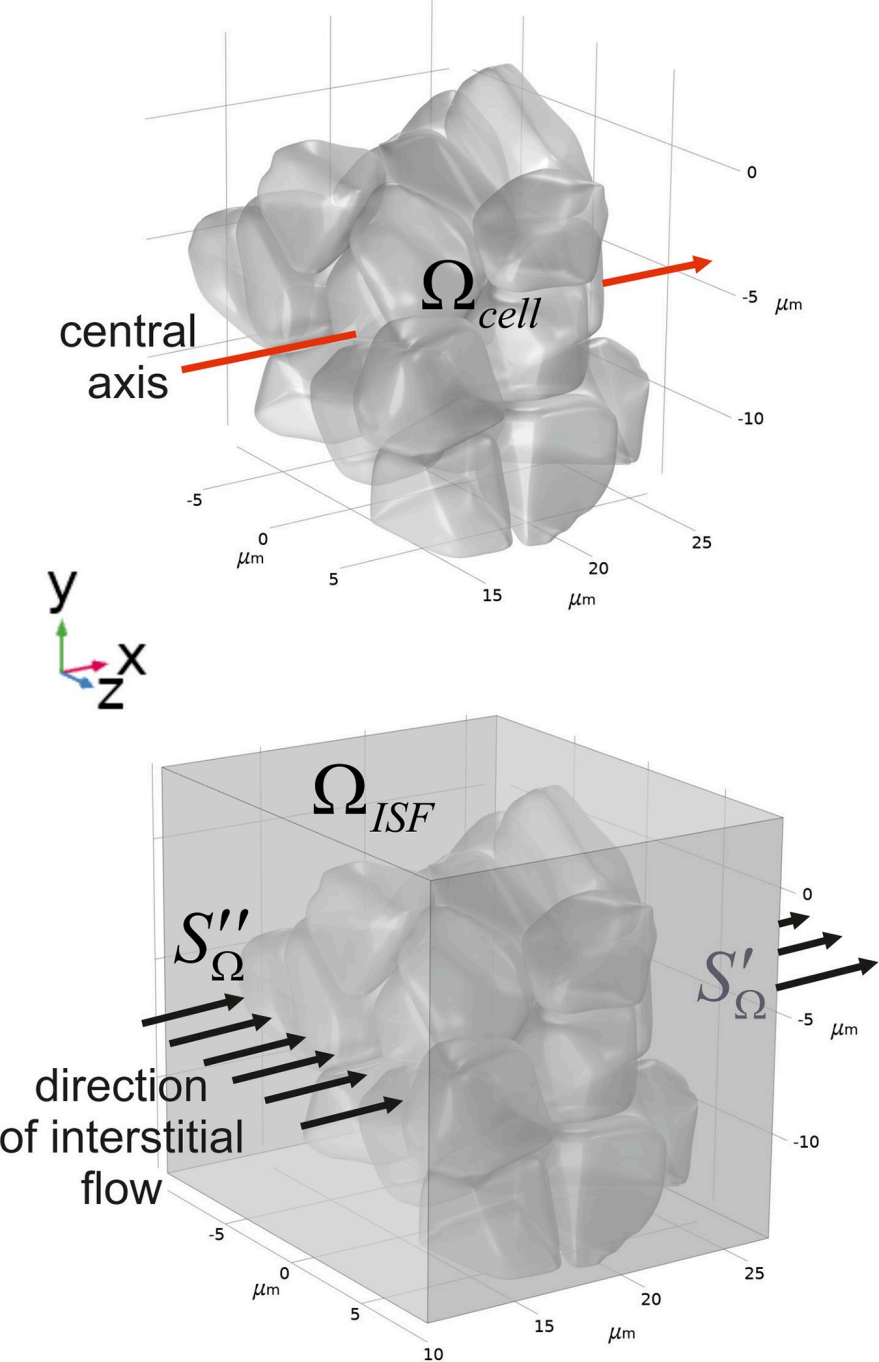
A multi-object simplified phantom of a local brain area. A limited area containing neurons and astrocytes is approximated by the set of 26 non-uniform objects, which simulate cell bodies and combinations of incoming dendrites and axons. The set of the bodies is formed as a bunch, where two central objects are surrounded by other ones (A). The character size of the objects is around 5 μm, and the distance between the surfaces of neighbors varied with a median of 8 nm [55]. The central axis is a line that penetrates the phantom at the level of y = -5μm (a red arrow). It lies in the central XY plane. The considered set of bodies is placed into parallelepiped 16×17×19 μm (B). ISF flow is constant, and it has an orthogonal direction to the YZ plane (B, black arrows). The production and consumption of H_2_O_2_ are localized into the objects.

Although the initial, primary death signals, i.e., those that induce NMDA receptor-mediated death and oxidative stress, may be localized in a narrow area, the further development of glutamate excitotoxicity seems to be essentially spatially dependent. In particular, it can necessarily mediate through Connexin 36-containing gap junctions [50]. Indeed, the metabolism of ROS has been accurately described to be aberrant in many neurodegenerative diseases [51]. Due to a lofty level of oxidative reaction with a single electron transfer, the respiratory chain in the inner mitochondrial membrane seems to be the main ROS production source. Nevertheless, it appears under very high rates of H_2_O_2_ scavenging by brain mitochondria [52], which can be as high as 100 times more than the highest rate of experimentally measured brain ROS generation [53]. This essential difference between the metabolic fluxes causes doubts about the ground hypothesis of ROS source in nervous tissue. Recently, Starkov et al. have shown that mitochondria are neither a sink nor a source of H_2_O_2_ but can function as both at the same time, efficiently stabilizing exogenous H_2_O_2_ concentration at a level directly proportional to the ratio of the H_2_O_2_ generation rate to the rate constant of the first order scavenging reaction [54]. Despite the balance of production and consumption fluxes, the question about possible ROS distribution still arises. It may be caused by both diffusion and convection of the species away from the local area of their generation. A local virtual area of brain tissue has been considered to analyze the possibility of increased hydrogen peroxide level appearance. It is approximated by a set of non-uniform 3D objects, which is designed in a manner when two central bodies are surrounded by other ones (see Fig 8).

**Fig 9:**
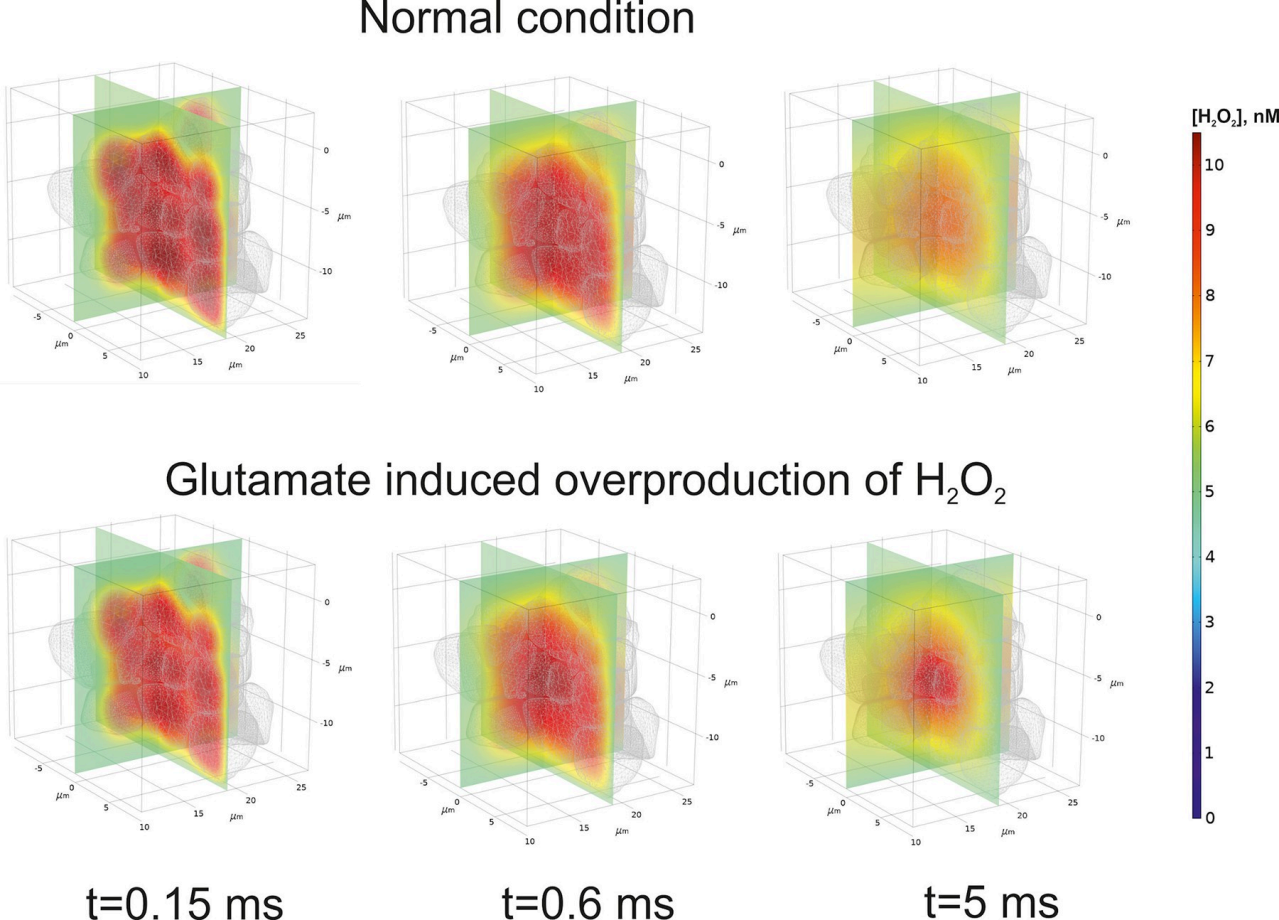
Numerical approximation of hydrogen peroxide concentration in a local brain area. Time dependent gradients of H_2_O_2_ concentration are represented in the two orthogonal planes cross the phantom in the central position. A mesh contour objects are also drawn but a front quarter is omitted for a clear visualization. The distributions are represented for several time points describing both initial and pre-steady state conditions. The normal condition (upper panel) means that the rate of H_2_O_2_ production in all objects in the phantom is physiological. Glutamate induction of hydrogen peroxide overproduction indicates that a high rate appears in the two central objects. The surrounding objects keep a normal rate of production.

Under such a condition, we suppose that glutamate initiates increased hydrogen peroxide production in the central core of mentioned two bodies. The other objects keep the production of H_2_O_2_ at the normal level. The flux defining the changes of hydrogen peroxide point concentration is the sum of production and consumption net rates (see part II of Models). It is assumed that the final summand will be positive because the cells have a non-zero level of ROS production. The results of numerical modeling are shown in Fig 9.

**Fig 10:**
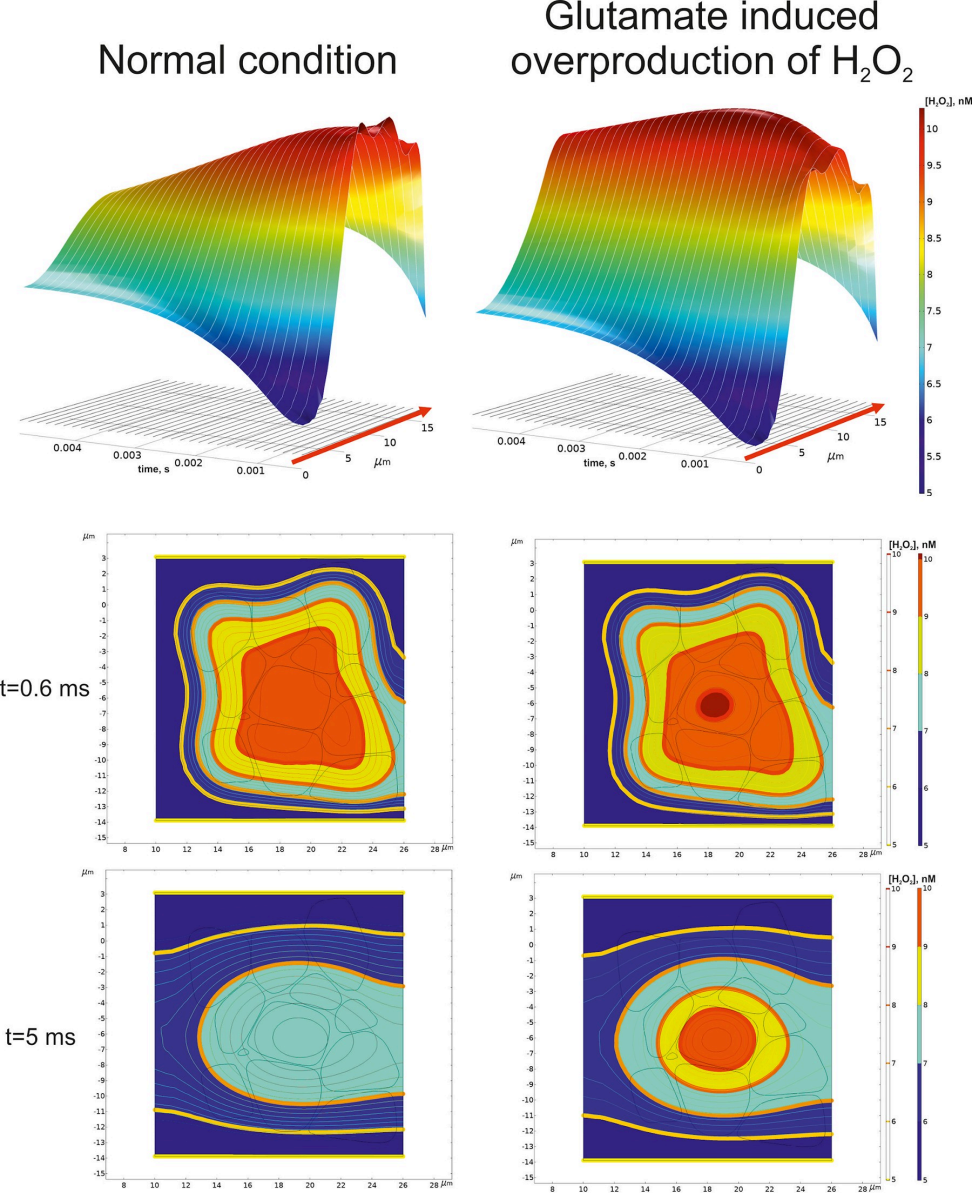
Spatial-time dependence of hydrogen peroxide concentration alone the central axis of the phantom. A 3D surface dependence of H_2_O_2_ concentration on time and coordinate is represented in the top for a normal condition (left) and a glutamate-induced overproduction(right). The red arrow indicates the direction of the center axis shown in Fig 8. The central XY plane with iso-lines of H_2_O_2_ concentrations and corresponding filled areas is shown below. The corresponding time moments are indicated in the left column.

Initially, the spatial distribution of hydrogen peroxide seems to be similar for normal and high production fluxes. At the first moment of time concentration of H_2_O_2_ is equal to 5 and 10 nM in ISF and cell space, respectively (see part II of Models). This difference approximates the gradient by a kink like function which is further smoothed by diffusion and convection. Due to only 4.8% of the considered phantom volume has got an increased production, the gradients near the objects are akin. However, an essential difference appears when the system becomes close to a steady state condition. It is clearly seen for the partial example of axis-oriented concentration distribution (Fig 10). Under normal conditions, hydrogen peroxide concentration decreases slowly in time. There is a maximal amount in the central area, but the level is never more than an initial value for the objects. However, if the hydrogen peroxide production is induced by glutamate, the level of H_2_O_2_ will excess over the initial values (a dark red spot in the central area in the right draw). Then, the gradient goes down as like as the same one under normal conditions. Exceeding the initial level is approximately 2.5%. A slightly increased concentration of H_2_O_2_ is kept in the central area of glutamate-induced tissue even though the steady state condition is reached.

## Discussion

In the presented theoretical analysis we checked whether the hypothetical mechanism of complex II activation by glutamate underlies the glutamate effects on the RC, observed in [29]. The analysis has confirmed that glutamate, indeed, can provoke such significant changes in ROS production. The biochemical basis for the observed huge increase in ROS production rate is complex II activation by glutamate, according to the mechanism visualized in Fig 1.

A stepwise switch from low to high ROS production rate is an essential characteristic of glutamate-induced activation of ROS production. Simulation of this phenomenon was possible because the model admits multistationarity in the RC operation. According to the model, the RC can function in two different steady states, as Fig 4B–4D indicates. Various agents induce a switch between these steady states. The absence of additivity in the effects of two agents causing an increase in ROS production, glutamate and antimycin, is also consistent with the hypothesis of the RC bistability. Once the switch to the state of high ROS production has occurred, an additional agent cannot further increase the effect of the first one.

The detailed kinetic model presented here uncovers a novel role of the well-known phenomenon of complex II inhibition by oxaloacetate as a key factor in the dramatic increase of ROS induced by a rise in glutamate concentration in brain mitochondria. The simulations in the most detailed kinetic model of respiration that accounts for this inhibition have shown that the latter is an essential factor controlling respiration rate and ROS generation. The analysis has demonstrated that if glutamate attenuates oxaloacetate’s inhibition under the condition of high workload, the RC provides the necessary energy more effectively. However, suppose such an attenuation takes place under conditions of decreased workload. In that case, the increase of complex II activity can lead to the RC switch into an ineffective and primarily ROS generating state thus provoking oxidative stress with its pathological consequences.

The analyzed here details of the RC stimulation by glutamate and switch of the RC to an “inactive” state where ROS generation rate highly increases and provokes oxidative stress provides a new understanding of the processes underlying excitotoxicity. We believe that such a deep understanding of this phenomenon will be a stimulus towards a potential therapeutic approach.

One of the main regulatory aspects of a glutamate tissue overload is a physiological or a therapeutic ability to reduce its toxic effect on the brain tissue. In general, diminution of excitotoxicity is achieved by an acceleration of excess glutamate clearance. In particular, it can be mediated through ISF flow, which distributes a high glutamate concentration to be moved by a bulk flow of the vascular system. Nevertheless, it has been functionally estimated that glutamate transport across brain endothelial cells in physiological conditions is minimal [1]. However, when the extracellular concentration of glutamate increases in the brain, EAATs accumulate it into brain endothelial cells with further facilitated diffusion to the blood. Several experiments have demonstrated that acceleration of the trans-endothelial to vascular luminal efflux of glutamate may be an important mechanism to reduce the neurotoxic effects of glutamate [56]. Convection and diffusion are the essential processes for toxic chemical gradients as well. Analysis of the spatial-time distribution of hydrogen peroxide indicates that even a small area treated with glutamate can be a potential source of radicals. The balance between ISF and cell initial concentrations has been chosen to get a mean value of nearly 8 nM in the brain tissue [54]. Indeed, the considered initial value of 10 nM is well below toxic levels of H_2_O_2_ [57]. Nevertheless, it is remarkable that glutamate overproduction causes an increased hydrogen peroxide level exceeding the initial level. The relatively low increased percent of concentration should be explained by an especial feature of the local considered area. Indeed, the enhanced level of hydrogen peroxide production appears in the two central objects only. The surrounding area produces H_2_O_2_ at a normal rate. Due to the typical size of the object of 5 μm, this example has to be deemed as the narrowest possible localization of measurable hydrogen peroxide synthesis. In other dimensions of the glutamate induced area, the effect will be more significant. Moreover, a small number of neighbors enhances the role of convection. However, Jin et al. modeling results do not support a physiologically important role for local parenchymal convective flow in solute transport through brain extracellular space [58]. Extension of the phantom border and enlarge the number of the objects will result in a shift of the balance to diffusion. It should be stressed that in our model tensor of diffusion is reduced to a zero rank (see part II of Models, (11)) because a tortuosity of the medium is considered explicitly. The results of our modeling agree with the previous ones obtained for inside hydrogen peroxide diffusion in HeLa cells [59]. Showed the time and length scales for H_2_O_2_ diffusion after membrane permeation to be approximately 1ms and 4 μm, respectively.

The possibility to counteract with vitamin E the maladaptive morphological and functional changes of brain related to glutamate-mediated excitotoxicity, neuroinflammation, and oxidative stress is clearly shown in the kainic acid model of pharmacological excitotoxicity and epileptogenesis [60].

The risk of glutamate excitotoxicity can also be diminished by microRNA, which exerts its effects by changing the transcriptional level of glutamate receptors in post-ischemic stroke [61]. Resveratrol suppresses the increase of extracellular Glu levels induced by stretch injuries by enhancing the Glu transport, thus increasing Glu uptake, glutathione content, glutamine synthetase activity, and S 100B (a neurotrophic cytokine) secretion in cortical astrocyte cultures and C6 glioma cells [62].

Curiously, the inhibitor (antimycin) and activator (glutamate) produced similar effects on ROS production. However, these opposite extremes result in the same: almost complete reduction of ubiquinone pool and deficiency of oxidized form that participates in QH_2_ oxidation in complex III Q-cycle. Thus, glutamate’s effects are controversial: it activates electron flow, but this activation could lead to the RC inhibition at low energy demand. This controversial outcome underlies the glutamate effects in the cells.

In addition to the considered here direct activation of complex II, the critical reason for glutamate excitotoxicity is that glutamate can markedly activate NMDA receptors, and increase the concentration of intracellular calcium, which is a potent activator of many processes. Such an activation could lead to their dysregulation and finally induce neuronal death. However, activation of the receptors and final glutamate effect seems to be not unambiguously connected. For example, the noncompetitive NMDA receptor antagonist memantine can protect dissociated cortical neurons from glutamate excitotoxicity [63], but glutamate antagonists induce schizophrenia-like symptoms through modulation of the NMDA receptor [63]. It means that the pathophysiology of schizophrenia may similarly result from dysregulation of the NMDA receptor, and the clinical syndrome of such a pathology reflects simply the behavioral pattern resulting from generalized disruption of NMDAR-mediated neurotransmission within the brain [64]. Current proponents of the glutamatergic hypothesis postulate that hypofunctional NMDA receptors located on gamma-aminobutyric acid (GABA)–ergic inhibitory interneurons disinhibit pyramidal neurons, leading to a paradoxical increase in glutamatergic activity [65].

The observed acceleration of Ca^2+^ uptake ([29], Fig 4) indicates that glutamate accelerates electron flow through the RC. These data validate the mechanism of complex II activation by glutamate (Fig 6) implemented in the model. In this case, rapid oxidation of QH_2_, necessary to recover Δψ compensates the accelerated reduction of Q, induced by glutamate, and the switch to high ROS production does not occur. The uncoupling conditions are qualitatively similar. That is why uncoupling prevents the switch to an elevated ROS producing state.

Reproducing the experimental data observed in isolated mitochondria validated the proposed mechanism of complex II activation by glutamate and the idea of bistability of the RC operation. Having this validation, we extended the model to simulate glutamate transport into cells from extracellular space and into mitochondria and energetic metabolism starting from glucose. The simulations of in situ conditions have shown that an increased concentration of glutamate might occur in situations of excessive sustained stimulation. It may result in the effect observed in isolated mitochondria, i.e., switching to the ROS producing state and cellular energetics compromising. Such a switch of the RC operation triggered by glutamate may have significant physiological consequences.

## Conclusions

- The model, simulating experimental data here developed, describes qualitatively all the experimentally measured effects of glutamate on isolated mitochondria and validated the hypothesis that glutamate activates complex II by stimulating oxaloacetate utilization, thus lowering its inhibition on complex II.
- The model, simulating experimental data unveils that the indirect activation of complex II, resulting from glutamate’s attenuation of oxaloacetate’s inhibition, plays a key role on ROS production in the respiratory chain and the interaction of respiratory complexes in this process.
- The model’s analysis has shown that the respiratory chain’s functional structure, which implements Mitchell’s Q-cycle in complex III, is consistent with multistationarity and hysteresis.
- The stepwise activation of ROS production by glutamate results from the glutamate-induced switch from a branch of slow ROS production steady states to a different one of rapid ROS production.
- Simulation of in situ conditions has shown that glutamate, which diffuses from synapses into extracellular space, may stimulate the switch of mitochondria into the state of high ROS production under moderate workloads. The reason for such a switch is increasing QH_2_ concentration due to the increasing rate of its production by complex II, and, respectively, Q deficiency. Such a switch disturbs cellular energetics and leads to a drop in ATP levels.
- Under high workloads, glutamate stimulates respiration but not a switch to the state of high ROS production, because the produced QH_2_ is readily utilized due to increased demand in ATP synthesis.
- Glutamate induced overproduction of hydrogen peroxide results in detectable increase of H_2_O_2_ in the model reaction-convection-diffusion. This effect depends on the size of the considered area.

## Models

### I. A kinetic model of mitochondrial and cellular energy metabolism

The open-source software tool "Mitodyn" was used to analyze the mitochondrial and cellular energy metabolism dynamics. It is based on a kinetic model, i.e., a system of ordinary differential equations (ODE) that describes the processes briefly summarized in Fig 1 of S1 Text. The model simulates the dynamics of redox states of the respiratory chain (RC) complexes, glycolysis, Krebs cycle, oxidative phosphorylation, ATP consumption, and transport of some metabolites through the cellular and inner mitochondrial membranes. Accounting the details of neurotransmitter glutamate transport and metabolism, including malate—aspartate shuttle, allows using this model to analyze neuronal cell specificity. The software is freely available at https://github.com/seliv55/cell_mito. The metabolic reactions not directly involved in the respiratory electron transport but presented in the model are described in S1 Text.

The software simulates the time course of model variables: the concentrations of metabolites and redox states of respiratory complexes, mitochondrial and sarcolemmal membrane potentials. It shows in output some functions of state variables: reactive oxygen species (ROS) production separately in various sites of the RC. Each time derivative of ODE variables, is a sum of reaction rates where the given variable participates. A reaction rate is positive if the variable is a product and negative if it is a substrate. The model respects NADH and ATP generation stoichiometry in the simulated processes. Mitodyn enables continuous calculations of the system’s dependencies on model parameters to study its bifurcation characteristics.

The model is mainly designed to analyze rates of electron transport in the RC and related reactive oxygen species (ROS) generation. It accounts for the details of these processes, whereas the others are simplified. The model of the RC is represented by ~300 ODEs because we describe details of electron transport in respiratory complexes that consist of fixed in space electron carriers. In this case, an accurate description should include the redox states of whole complexes as variables. The number of such states is large, and the number of variables corresponds to it. Such a detailed description is necessary to model the details of ROS generation separately in various sites of the RC. It should be noted that the model contains much fewer parameters than equations because the same electron transport reactions for different redox states are described using the same rate constants [37]. In simulations of the experiments performed in the presence of rotenone, we did not account for ROS generation in complex I. It was shown that even with rotenone present, if NADH levels are sufficiently elevated, ROS can be generated at the FMN redox centers when hydrophilic quinones are used as electron acceptors [66, 67]. However, as stated in [67], it is not the case without artificially added hydrophilic quinones in the presence of rotenone. Therefore, in the model, we did not account for ROS generation in rotenone inhibited complex I.

**Fig 11:**
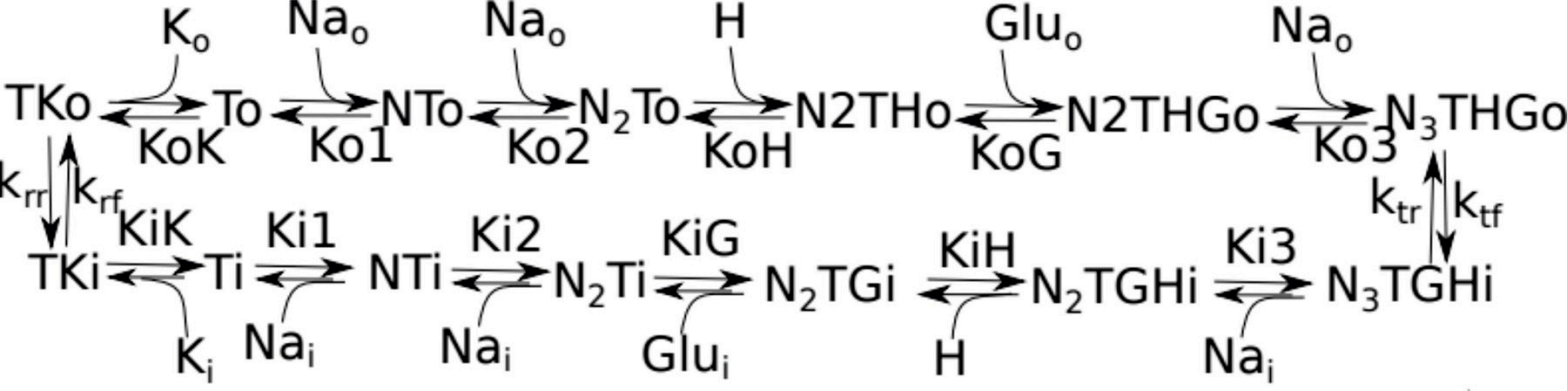
The scheme of glutamate transport implemented in the model. Outside Na+ (Nao) binds to the free transporter in the ‘outer’ configuration (T), accessible for binding outside ions. This binding facilitates the binding of glutamate (Gluo) and then *Na⁺* atoms again. The model assumes fast equilibrium in these steps. The transporter changes configuration (T’), facilitating dissociation of all bound Na+ ions and glutamate inside cells. *K⁺* binding facilitates a switch of the transporter to the outer structure and dissociation of bound *K⁺* outside.

The model accounts for the detail of neurotransmitter glutamate transport from extracellular space to study its interaction with intracellular energy metabolism. The steps of this transport accounted for in the model are indicated in Fig 11. As currently accepted, the transport cycle starts with sodium ion binding to the transporter (EAAT) [68, 69]. Then a second sodium ion is bound [70, 71]. Computational study shows that the most suitable glutamate clearance scenario is that the glutamate ion binds after the second sodium ion [24] to the protonated transporter [72]. Conformation change in the transport domain after glutamate binding facilitates the closing of extracellular gates [70]. The third sodium ion association stabilizes the transporter in closed configuration [73] to provide cross membrane movement of the EAAT transport domain. After the dissociation of the externally bound ions to the cytosol, which proceeds in reverse order, EAAT can bind *K⁺* ion. Then reverse conformation change transfers the bound *K⁺* outside the cell, thus terminating the transport cycle. This mechanism, outlined in [24], is implemented in our model (see S1 Text).

### II. Spatial-temporal gradients of hydrogen peroxide in a local phantom of a nervous tissue

Diffusion of hydrogen peroxide is considered in a cluster of non-uniform bodies with narrow sheets and tunnels between them. This cluster is placed into the parallelepiped as described above (Fig 9). A limited area containing neurons and astrocytes approximated by the set of 26 non-uniform objects, which simulate cell bodies and combinations of incoming dendrites and axons. Diffusion and convection of hydrogen peroxide in the phantom are combined with production of H_2_O_2_ in the objects. This production can be considered either as normal or as high depending on glutamate influence. Incoming flux through $S_{\Omega }^{\prime \prime }$ is described according to Danckwerts border condition. The outflow stream out through $S_{\Omega }^{\prime }$. For other sides of the parallelepiped the fixed concentration conditions have been chosen. For modeling of reaction-diffusion in neuropils, tortuosity of the medium is usually taken into account [74, 75]. However, we do not need to make assumption about porosity of the phantom because the complex form of the bodies/ISF space is considered explicitly. It results in reduction of diffusion tensor to a zero rank. The mathematical border problem with initial condition will have the following form:

$$\begin{array}{l} \frac{\partial c_{H_{2}O_{2}}(\vec{r},t)}{\partial t}=\nabla \cdot (D_{H_{2}O_{2}}\cdot \nabla c_{H_{2}O_{2}}(\vec{r},t))-{\vec{u}}_{ISF}\cdot \nabla c_{H_{2}O_{2}}(\vec{r},t)+J_{H_{2}O_{2}};\\ {c_{H_{2}O_{2}}(\vec{r},t)|}_{t=0}=\{\begin{array}{l} C_{H_{2}O_{2}}{}^{cell},\vec{r}\in \Omega_{\mathrm{cell}}\\ c_{H_{2}O_{2}}{}^{ISF},\vec{r}\in \Omega_{ISF} \end{array};\;J_{H_{2}O_{2}}=\{\begin{array}{l} J\neq 0,\vec{r}\in \Omega_{\mathrm{cell}}\\ 0,\vec{r}\in \Omega_{ISF} \end{array};\\ \vec{n}\cdot (\vec{J}+\vec{u}\cdot c_{H_{2}O_{2}}(\vec{r},t))=\vec{n}\cdot ({\vec{u}}_{ISF}\cdot c_{H_{2}O_{2}}{}^{ISF}),\vec{r}\in S_{\Omega }^{\prime \prime };\\ \vec{n}\cdot (D_{H_{2}O_{2}}\cdot \nabla c_{H_{2}O_{2}}(\vec{r},t))=0,\;\vec{r}\in S_{\Omega }^{\prime };\\ c_{H_{2}O_{2}}(\vec{r},t)=c_{H_{2}O_{2}}{}^{ISF},\vec{r}\notin S_{\Omega }^{\prime \prime }\vee S_{\Omega }^{\prime }; \end{array}$$ (11)

The numerical solution of (11) was obtained using COMSOL Multiphysics ver. 5.5. The parameters used in the modelling of convectional diffusion have been represented in Table 1.

**Table 1:** The diffusion property of the medium.

Symbol	Parameter	Value	Source
$D_{H_{2}O_{2}}$	Hydrogen peroxide diffusion coefficient	1.83×10^−9^ m^2^/s	[76]
$\left|{\vec{u}}_{ISF}\right|$	Brain extra cellular fluid bulk flow velocity (ISF flow)	5.0×10^−7^ m/s	[77]
$J_{H_{2}O_{2}}$	Normal H_2_O_2_ production	5.05 μM/min	[29]
	High H_2_O_2_ production stimulated by glutamate	106 μM/min	
$C_{H_{2}O_{2}}{}^{cell}$	Initial concentration of hydrogen peroxide in the cell bodies	10 nM	[57]
$C_{H_{2}O_{2}}{}^{ISF}$	Initial concentration of hydrogen peroxide in ISF	5 nM	[54]

The diffusion coefficient for H_2_O_2_ is used in (11) as a constant parameter describing isotropic symmetric diffusion. The velocity of ISF flow is represented as a mean module value. The direction of the vector is indicated in Fig 8.

## Supporting information

S1 Text A kinetic model of cell energy metabolism without the part of the mitochondrial respiratory chain. The model implementation of the Krebs cycle, glycolysis, malate-aspartate shuttle and glutamate transport reactions are described in detail.
(PDF)
