# Supplementary material for: Unveiling a key role of oxaloacetate-glutamate interaction in regulation of respiration and ROS generation in nonsynaptic brain mitochondria using a kinetic model
Source: PLoS One. 2021 Aug 3;16(8):e0255164. doi: 10.1371/journal.pone.0255164 (PMC8330910; doi:10.1371/journal.pone.0255164)
Supplement: S1 Text — The model implementation of the Krebs cycle, glycolysis, malate-aspartate shuttle and glutamate transport reactions are described in detail. (PDF) [file pone.0255164.s001.pdf]

Implemented in the model metabolic reactions that are not directly involved into the respiratory electron transport.

Fig 1 briefly summarizes the processes described by the model. The model simulates the dynamics of redox states of the respiratory chain (RC) complexes, glycolysis, Krebs cycle. Accounting for detailed neurotransmitter glutamate transport and metabolism, including the malate - aspartate shuttle, allows application of this model to an analysis of specifically neuronal processes. The state variables of the ODEs system correspond to the concentrations of substrates and products. The equations are expressions for time derivatives of the state variables. Each time derivative of a variable is expressed as the sum of rates of reactions where the given variable participates. The rate is positive if the variable is a product of the reaction and negative if it is a substrate.

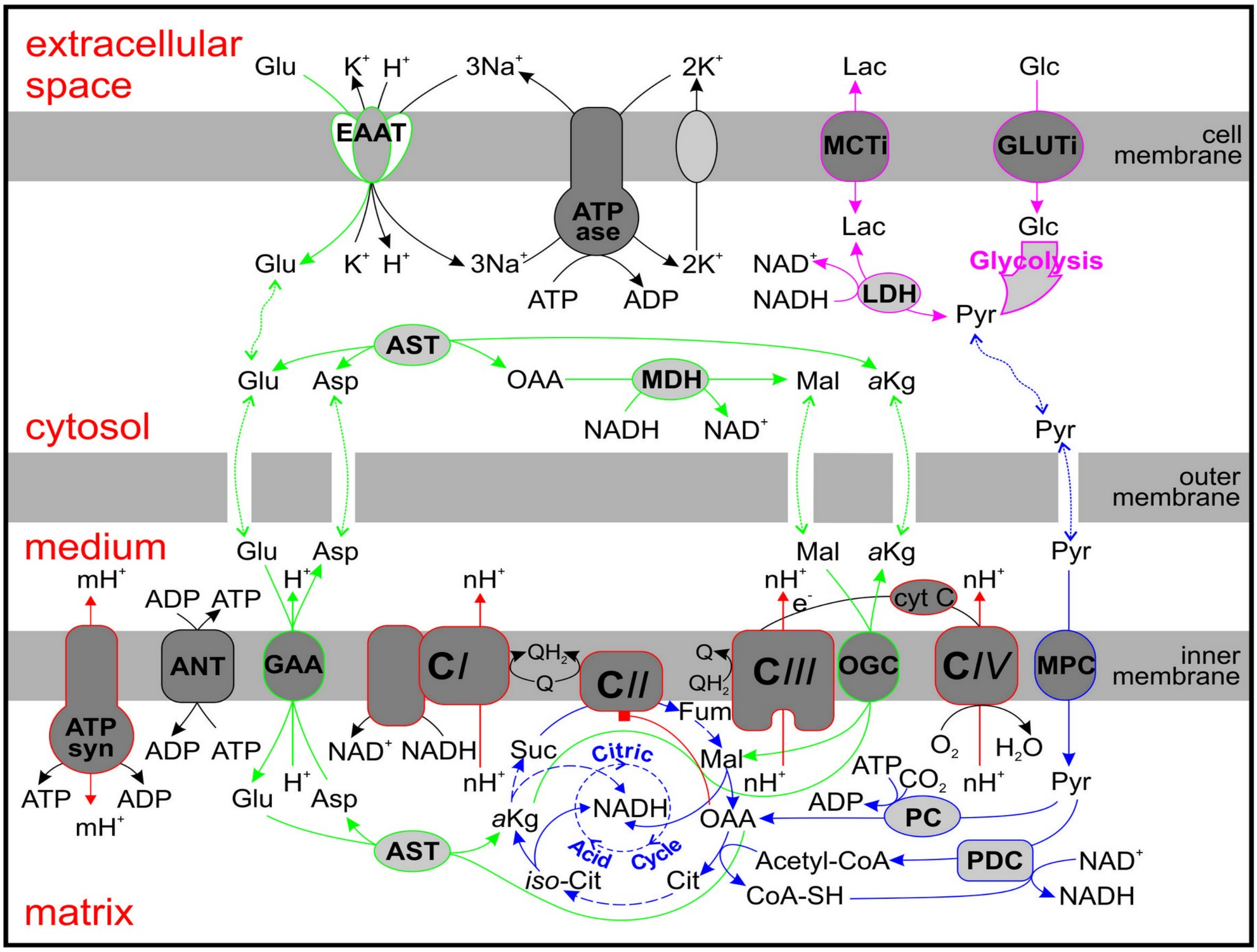

Fig 1. A summary of the processes represented in the model. Metabolites, described in the model, are labeled by letters. Arrows represent reactions converting or transporting respective metabolites or ions. Blue arrows indicate the Krebs cycle reactions, green arrows – malate – aspartate shuttle, violet arrows – glycolysis. The red line ended by square shows complex II inhibition by oxaloacetate. Black arrows indicate other processes. “CI” – ”CIV” in circles label respiratory complexes I up to IV. Cofactors NADH, FAD, ATP are not shown in some reactions for simplicity. Other explanations are in the text. Abbreviations: aKg, α-ketoglutarate; Asp, aspartate; CI, CII, CIII, CIV, respiratory complexes I, II, III, IV; Cit, citrate; Fum, fumarate; Glu, glutamate; Glc, glucose; Lac, lactate; Mal, malate; OAA, oxaloacetate; Pyr, pyruvate; RC, respiratory chain; ROS, reactive oxygen species; Suc, succinate.

The model is designed to analyze in detail electron transport and ROS generation in the RC but other processes are simplified. Fig 1 denotes the state variables corresponding to metabolite concentrations in the model. Glycolysis is lumped into one reaction representing the conversion of glucose into pyruvate and lactate with the respective stoichiometries of NADH and ATP.

Simplified representation of glycolysis in the model. The glycolytic pathway is lumped from glucose to pyruvate in one step, Gluc → pyr. The rate of this reaction is

$v_{gl} = k_{gl}\times(1\text{-}C_{pyr})$, (1)

where C_{pyr} is a dimensionless pyruvate concentration that changes in the interval (0,1), k_{gl}=0.0375 s^{-1} is the rate constant.

The contribution of glycolysis to the pyruvate, NAD and ATP concentrations is given by:

$d(C_{pyr})_{gl}/d(t) = v_{gl}$

$d(C_{NADc})_{gl}/d(t) = -\ v_{gl}$ (2)

$d(C_{ATP})_{gl}/d(t) = 2v_{gl}$

The model accounts for the lactate dehydrogenase (LDH) step, pyr ↔ lac, as

$v_{LDH}= k_{LDH}\times(C_{pyr}\times C_{NADHC} - C_{lac}\times C_{NADc})$ (3)

Here k_{LDH} = 7.5 s^{-1} is the rate constant, C_{lac}, C_{NADHc}, and C_{NADc} are the pyruvate, lactate, cytosolic NADH and NAD concentrations, respectively.

The contribution of lactate dehydrogenase to the levels of pyruvate, lactate and cytosolic NAD is given by

$d(C_{pyr})_{LDH}/d(t) = -v_{LDH}$

$d(C_{lac})_{LDH}/d(t) = v_{LDH}$ (4)

$d(C_{NADc})_{LDH}/d(t) = v_{LDH}$

The sum of NADH and NAD concentrations in cytoplasm is conserved, $C_{NADHc} + C_{NADc} = 17$ nmol/mg.

An exchange between outer and inner lactate is described as

$v_{ex}= k_{ex}\times(C_{laco} - C_{lac})$ (5)

Here k_{ex} = 7.5 s^{-1} is the rate constant, C_{lac} is the inner lactate concentration, C_{laco} is the constant outer lactate concentration, which is set to 0 for the presented simulations.

A contribution of this process to the lactate pool is

$d(C_{lac})_{ex}/d(t) = v_{ex}$ (6)

Pyruvate feeds the Krebs cycle through the pyruvate dehydrogenase reaction, which in the model is combined with ***citrate synthesis***. Due to this combination pyruvate and mitochondrial NAD (C_{NAD}) are implemented as substrates of this process as well as oxaloacetate (C_{OAA}).

$v_{CS} = k_{CS}\times C_{OAA}\times C_{pyr}\times C_{NAD}$ (7)

Here k_{CS} = 7.5 $(nmol/mg)^{-1}\times s^{-1}$ is the rate constant, A contribution of this process to the levels of oxaloacetate, pyruvate, NAD, and citrate (C_{cit}) are

$d(C_{OAA})_{cs}/d(t) = -\ v_{CS}$

$d(C_{pyr})_{cs}/d(t) = -\ v_{CS}$ (8)

$d(C_{NAD})_{cs}/d(t) = -\ v_{CS}$

$d(C_{cit})_{cs}/d(t) = v_{CS}$

The sum of NADH and NAD concentrations in mitochondria is conserved, $C_{NADH} + C_{NAD} = 17$ nmol/mg.

Transformation of citrate into α-ketoglutarate in the model is lumped into one step, as well as the subsequent conversion of α-ketoglutarate into succinate.

$v_{citakg} = k_{citakg} \times C_{NAD} \times C_{cit}$ (9)

Here k_{citakg} = 2250 $(nmol/mg)^{-1} \times s^{-1}$. This process contributes to the total derivatives of mitochondrial NAD, citrate and α-ketoglutarate.

$d(C_{NAD})_{citakg}/d(t) = - v_{citakg}$

$d(C_{cit})_{citakg}/d(t) = - v_{citakg}$ (10)

$d(C_{akgm})_{citakg}/d(t) = v_{citakg}$

Similarly, the model represents α-ketoglutarate conversion into succinate as one step process.

$v_{akgsuc} = k_{akgsuc} \times C_{NAD} \times C_{akgm}$; (11)

Here k_{akgsuc} = 2250 $(nmol/mg)^{-1} \times s^{-1}$. This process contributes to the total derivatives of NAD, α-ketoglutarate and succinate (C_{suc}).

$d(C_{NAD})_{akgsuc}/d(t) = - v_{akgsuc}$

$d(C_{akgm})_{akgsuc}/d(t) = - v_{akgsuc}$ (12)

$d(C_{suc})_{akgsuc}/d(t) = v_{akgsuc}$

Similarly, the model accounts for succinate oxidation and subsequent fumarate conversion into malate as a one-step reaction inhibited by oxaloacetate [1]. Succinate oxidation by respiratory complex II initiates electron transport, that the model takes into account in detail (see Text S2). The subsequent malate dehydrogenase reaction along with the malate – aspartate shuttle are also part of the model.

The ***malate – aspartate shuttle*** is represented by two enzymatic reactions that are the same in the cytoplasm and mitochondria but proceeding in opposite directions. Aspartate aminotransferase in cytoplasm and mitochondria reversibly converts glutamate and oxaloacetate into α-ketoglutarate and aspartate. Malate dehydrogenase reversibly converts malate into oxaloacetate respecting the stoichiometry of NADH in cytosol and mitochondria. Two transport processes are part of the malate-aspartate shuttle, one representing the transport of cytoplasmic glutamate into mitochondria in exchange for mitochondrial aspartate, and the other the exchange of cytoplasmic malate for mitochondrial α-ketoglutarate.

The expression for the rate of malate dehydrogenase (MDH) reaction in cytosol is

$v_{MDHc} = k_{MDHc} \times C_{NADHc} \times C_{OAAc}$; (13)

Here k_{MDHc} = 75 $(nmol/mg)^{-1} \times s^{-1}$. The contribution of MDH reaction to the derivatives of cytosolic NAD, oxaloacetate, malate is

$d(C_{NAD})_{MDHc}/d(t) = v_{MDHc}$

$d(C_{OAAc})_{MDHc}/d(t) = - v_{MDHc}$ (14)

$d(C_{malc})_{MDHc}/d(t) = v_{MDHc}$

Malate dehydrogenase (MDH) reaction is favored in the direction of NADH oxidation. Equilibrium constants for the isolated malate dehydrogenase, K=[OAA]×[NAD]/([malate]×[NADH]), is $(2.86 \pm 012) \times 10^{-5}$ [Guynn 1973]. This value signify that in mitochondria MDH should produce malate. Since it produces oxaloacetate, we assumed that a kind of channeling exist in intact structure of mitochondria, so that inside it local malate concentration is at least five orders higher that that of oxaloacetate. Such kind of channeling would force the enzyme to produce oxaloacetate. We do not prove this assumption because the mechanism of MDH reaction is out of scope for this contribution, but it gives some reason for simulating Krebs cycle in forward direction. Thus, in mitochondria the net flux of MDH reaction is

$v_{MDH} = k_{MDH} \times C_{NAD} \times C_{mal}$; (15)

Here k_{MDH} = 75 (nmol/mg)$^{-1}$×s^{-1}. The contribution of MDH reaction to the derivatives of mitochondrial NAD, oxaloacetate and malate is

$d(C_{NAD})_{MDH}/d(t) = - v_{MDH}$

$d(C_{OAA})_{MDH}/d(t) = v_{MDH}$ (16)

$d(C_{mal})_{MDH}/d(t) = - v_{MDH}$

Aspartate aminotransferase (AT) reaction: OAA + glu ↔ asp + akg

In mitochondria:

$v_{AT} = k_{ATf}\times C_{OAA}\times C_{glu} - k_{ATr}\times C_{asp}\times C_{akg}$; (17)

Here k_{ATf} = 1100 (nmol/mg)$^{-1}$×s^{-1}, k_{ATr} = 750 (nmol/mg)$^{-1}$×s^{-1}.

$d(C_{OAA})_{AT}/d(t) = - v_{AT}$

$d(C_{glu})_{AT}/d(t) = - v_{AT}$

$d(C_{asp})_{AT}/d(t) = v_{AT}$ (18)

$d(C_{akg})_{AT}/d(t) = v_{AT}$

In cytosol:

$v_{ATc} = k_{ATf}\times C_{OAAc}\times C_{gluc} - k_{ATr}\times C_{aspc}\times C_{akgc}$; (19)

$d(C_{OAAc})_{AT}/d(t) = - v_{ATc}$

$d(C_{gluc})_{AT}/d(t) = - v_{ATc}$

$d(C_{aspc})_{AT}/d(t) = v_{ATc}$ (20)

$d(C_{akgc})_{AT}/d(t) = v_{ATc}$

Transport: exchange of mitochondrial α-ketoglutarate for cytosolic malate and cytosolic α-ketoglutarate for mitochondrial malate

$v_{mal/akg} = k_{mal/akg}\times(C_{malc}\times C_{akg} - C_{mal}\times C_{akgc})$; (21)

Here $k_{mal/akg}$ = 7.5 (nmol/mg)$^{-1}$×s^{-1},

$d(C_{akgc})_{mal/akg}/d(t) = v_{mal/akg}$

$d(C_{mal})_{mal/akg}/d(t) = v_{mal/akg}$

$d(C_{akg})_{mal/akg}/d(t) = - v_{mal/akg}$ (22)

$d(C_{malc})_{mal/akg}/d(t) = - v_{mal/akg}$

Transport: exchange of mitochondrial OH^- for cytosolic glutamate and cytosolic OH^- for mitochondrial glutamate

$v_{glu/asp} = k_{glu/asp}\times(C_{malc}\times C_{akg} - C_{mal}\times C_{akgc})$; (23)

Here $k_{glu/asp}$ = 150 (nmol/mg)$^{-1}$×s^{-1},

$d(C_{malc})_{glu/asp}/d(t) = - v_{glu/asp}$

$d(C_{akg})_{glu/asp}/d(t) = - v_{glu/asp}$ (24)

$d(C_{mal})_{glu/asp}/d(t) = v_{glu/asp}$

$d(C_{akgc})_{glu/asp}/d(t) = v_{glu/asp}$

Transport: exchange of mitochondrial aspartate for cytosolic glutamate and cytosolic aspartate for mitochondrial glutamate

$v_{glu/OH} = k_{glu/OH}\times(C_{gluc} - C_{glu})$; (25)

Here $k_{glu/OH}$ = 3.75 s^{-1},

$d(C_{gluc})_{glu/OH}/d(t) = - v_{glu/OH}$

$d(C_{glu})_{glu/OH}/d(t) = v_{glu/OH}$ (26)

The model accounts for the NADH and ATP generation stoichiometry in glycolysis and the Krebs cycle (although not shown in Fig 1). The transformation of one glucose molecule to pyruvate produces two NADH and two ATP. Pyruvate conversion into lactate consumes one NADH. Since citrate synthesis in the model includes the pyruvate dehydrogenase step, this reaction produces

NADH. Each of the transformations, citrate into α-ketoglutarate, the latter into succinate, and malate into oxaloacetate, produces NADH.

The model accounts for neurotransmitter ***glutamate transport*** from extracellular space to study its interaction with intracellular energy metabolism. Fig 1 schematically indicates glutamate transport together with other linked processes.

The scheme of glutamate transport shown in Fig 11 of the main text. The model accounts that the conformation changes linked with ions transition is the rate-limiting steps in the transport. Rate constants of these steps depend on the difference of the electric potentials between the sides of the outer cellular membrane (Vm):

$k_t(Vm) = k_t(0)\cdot exp(z_t \cdot F\cdot Vm/(R\cdot T))$ (27)

$k_r(Vm) = k_r(0)\cdot exp(z_r \cdot F\cdot Vm/(R\cdot T))$ (28)

Here, F is the Faraday constant, T the temperature, R the gas constant, z_t =3 and z_r =-1 are the number of elementary charges traversed the membrane in one cycle, which sign is positive if the direction of transport coincides with the direction of force from the transmembrane electric field and negative if the directions are opposite.

The rates of forward glutamate transport and K+ efflux coupled with return to the initial conformation are:

$v_t = k_{tf}(Vm)\cdot[N_3THGo] - k_{tr}(Vm)\cdot[N_3THGi]$ (29)

$v_r = k_{rf}(Vm)\cdot[TKi] - k_{rr}(Vm)\cdot[TKo]$ (30)

Assuming fast equilibrium between all outer and inner states allows calculating the concentrations [N3THG] and [T'K] solving respective systems of equations. The following system corresponds to the fast equilibrium between the outer forms.

KoK = To*K/TKo
Ko1 = T*Na/NT
Ko2 = NT*Na/N2T
KoH = N2T*Ho/N2TH (31)
Kog = N2TH*Glu/N2THG
Ko3 = N2THG*Na/N3THG
To =TK + T + NT + N2T + N2TH + N2THG + N3THG

The solution gives quasi equilibrium concentrations of all outer forms. In particular, the concentrations of TKo and N_3THGo, which are needed for the calculation of the rates in Eqs (29) and (30), are:

TKo = (K*Ko1*Ko2*Ko3*KoH*Kog*T0o)/(KoK*(Glu*Ho*Na^3+(Ho*(Ko3*Kog+Glu*Ko3)
+Ko3*KoH*Kog)*Na^2 +Ko2*Ko3*KoH*Kog*Na+Ko1*Ko2*Ko3*KoH*Kog)
+K*Ko1*Ko2*Ko3*KoH*Kog)

N_3THGo = (Glu*Ho*KoK*Na^3*T0o)/(KoK*(Glu*Ho*Na^3
+ (Ho*(Ko3*Kog+Glu*Ko3)+Ko3*KoH*Kog)*Na^2 +Ko2*Ko3*KoH*Kog*Na
+Ko1*Ko2*Ko3*KoH*Kog) + K*Ko1*Ko2*Ko3*KoH*Kog)

Here T0o is the sum of concentrations of all outer forms. With given rate constants this solution gives the concentrations of outer states of the transporter as functions of outside K^+ , Na^+, H^+ and glutamate.

Similarly, the following system corresponds to the fast equilibrium between the inner forms.

KiK = Ti*K/TKi
Ki1 = Ti*Na/NTi

Ki2 = NTi*Na/N2Ti

KiG = N2Ti*Glu/N2TGi (32)

KiH = N2TGi*Hi/N2TGHi

Ki3 = N2TGHi*Na/N3TGHi

Ti = TKi + T0i + NTi + N2Ti + N2TGi +N2TGHi + N3TGHi

The solution gives quasi equilibrium concentrations of all inner forms. In particular, the concentration of Tki and N_3THGi, which are needed for the calculation of the rates in Eqs (29) and (30), are:

TKi = (K*Ki2*Ki3*KiG*KiH*KiN*T0i)/(KiK*(Glu*Hi*Na^3+(Glu*(Ki3*KiH+Hi*Ki3) +Ki3*KiG*KiH)*Na^2+Ki2*Ki3*KiG*KiH*Na+Ki2*Ki3*KiG*KiH*KiN) +K*Ki2*Ki3*KiG*KiH*KiN)

N3TGH = (Glu*Hi*KiK*Na^3*T0i)/(KiK*(Glu*Hi*Na^3+ (Glu*(Ki3*KiH+Hi*Ki3)+Ki3*KiG*KiH)*Na^2 +Ki2*Ki3*KiG*KiH*Na+Ki2*Ki3*KiG*KiH*KiN) +K*Ki2*Ki3*KiG*KiH*KiN)

Here T0i is the sum of concentrations of all inner forms. With given rate constants this solution gives the concentrations of inner states of the transporter as functions of outside K^+ , Na^+, H^+ and glutamate.

The dynamics of T0i and T0o are described by the following equations:

$d(T0i)/dt = v_t - v_r$ (33)

Here v_t and v_r are the rates of transition between outer and inner forms described by Eqns (29) and (30). Since the total amount of the transporters T0 is conserved, T0o =T0 – T0i.

In steady state $v_t = v_r$ and T0i not changed, but glutamate transport continues with the rate v_t , Na^+ is transported into cytosol with the rate $3v_t$ and K^+ with the rate v_r.

The values of rate constants verified in [2] and used in the model are shown in Table 1.

Table 1. Equilibrium constants of the reactions indicated in Fig 11 of the main text.

Elementary reaction	Keq
To + Na ↔NTo	Ko1=0.25 mol/l
NTo + Na ↔ N_2To	Ko2=0.25 mol/l
N_2To + H ↔ N_2THo	KoH=1.2 $*10^{-9}$ mol/l
N_2THo + Glu ↔ N_2THGo	Kog=4.4$*10^{-5}$ mol/l
N_2THGo + Na ↔ N_3THGo	Ko3=0.1 mol/l
N3THGo ↔ N_3THGi	Koi=1.1
N_3THGi ↔ N_2THGi + Na	Ki3=50 l/mol
N_2THGi ↔ N_2TGi + H	KiH=1.5$*10^7$ l/mol
N_2TGi ↔ N_2Ti + Glu	Kig=1.3$*10^2$ l/mol
N_2Ti ↔ NTi + Na	Ki2=4$*10^2$ l/mol
NTi ↔ Ti + Na	Ki1=2.5$*10^2$ l/mol
Ti + K ↔ TiK	Kik=1$*10^{-3}$ mol/l
TiK ↔ ToK	Kio=10
ToK ↔ To + K	Ko=0.08 l/mol

In nervous tissue, glutamate can escape from the synaptic cleft, generating extra-synaptic glutamate dynamics [3]. This process is referred as ***Glutamate spillover.*** Mean glutamate concentration in extracellular space can reach 10 μM after a series of 5 stimulations [3], and twice higher after 50 stimuli [4]. The model simulates this process as glutamate flux into extracellular space proportional to a difference between the glutamate actual concentrations in the synapse (averaged in time) and

extracellular space.The model accounts for the diffusion of glutamate from the synaptic cleft into extracellular space as follows

$J_{Glu}=k_{Glu}\cdot(Glu_s-Glu_o)$ (34)

$k_{Glu}=7.5\ s^{-1}$

$d(C_{Gluo})_{GluDif}/d(t) = -\ J_{Glu}$ (35)

The K^+ concentration is ~140 mM in cytosol and ~5 mM in extracellular space. The model assumes some permeability for K^+ ions through the cell membrane that by translocation following their concentration gradient raises the cellular membrane potential Counteracting further efflux of K^+. The model accounts for this electrochemical equilibrium that defines ***Membrane potential due to K+ gradient and K+ conductance.***

The model accounts for K+ concentration ~140 mM in cytoplasm and ~5 mM in extracellular space. K^+ is permeable and translocates according to the concentration gradient which raises the membrane potential impeding further efflux of K^+. The model accounts for this electrochemical equilibrium using Goldman equation:

$J_k=P\cdot\mu\cdot(K_i-K_o\cdot exp(\mu))/(1-exp(\mu))$ (36)

Here J_k is the K^+ flux, $P=0.75\ s^{-1}$ is permeability coefficient, $\mu=Vm\cdot F/(R\cdot T)$.

J_k affects the inside and outside K+ concentrations and the membrane potential (Vm).

$d(C_{Ko})_{Kc}/d(t) = J_k$

$d(C_{Ki})_{Kc}/d(t) = -\ J_k$ (37)

$d(Vm)_{Kc}/d(t) = 2\cdot J_k\cdot F/C$

At inside K^+ of 140 mM and outside 5 mM, the calculated steady state Vm is 80 mV.

Na^+/K^+-ATPase uses the energy of ATP to export Na^+ and import K^+ through the cell membrane against their gradients. For every ATP molecule that it hydrolyzes, three Na^+ are exported, and two K^+ are imported. Net export is a single positive charge per pump cycle.

The model represents this process in simplified form as follows:

Na^+/K^+-ATPase:

$J_{NaK}=k_{NaK}\cdot ATP\cdot Na_i\cdot K_o$ (38)

$k_{NaK}=0.0075\ (nmol/mg)^{-1}\times s^{-1}$

Other ATPases:

$J_{ATPase}=k_{ATPase}\cdot ATP$ (39)

$k_{ATPase}=0.75\ s^{-1}$

$d(C_{ATP})_{Na/K}/d(t) = -\ J_{NaK}$

$d(C_{ATP})_{ATPase}/d(t) = -\ J_{ATPase}$

$d(C_{ADP})_{Na/K}/d(t) = J_{NaK}$

$d(C_{ADP})_{ATPase}/d(t) = J_{ATPase}$ (40)

$d(C_{Ki})_{Na/K}/d(t) = 2\cdot J_{NaK}$

$d(C_{Ko})_{Na/K}/d(t) = -\ 2\cdot J_{NaK}$

$d(C_{Nai})_{Na/K}/d(t) = -\ 3\cdot J_{NaK}$

$d(C_{Nao})_{Na/K}/d(t) = 3\cdot J_{NaK}$

$d(C_{Vm})_{Kc}/d(t) = 2\cdot J_{NaK}\cdot F/C$

ATP synthase implemented in the model accounts for the stoichiometry of proton translocation by the RC. Oxidation of substrates in complexes I, III, and IV is coupled with proton translocation from the matrix into the intermembrane space. The model accounts for complex I translocation of 4

H^+ per NADH molecule oxidized [5], and complex III translocation of 2 H^+ per QH_2 oxidized. Two protons are released from QH_2 to the outer side [6], and complex IV translocates 2 H^+ per 2 cytochromes c oxidized [7]. Protons and other ions translocation across the inner mitochondrial membrane change the transmembrane electric potential $\Delta\psi$ which is used to drive ATP synthesis. The model accounts for the translocation of 4 H^+ back to the mitochondrial matrix per one ATP synthesized from ADP and inorganic phosphate [8]. It describes this process in simplified form taking into account a dependence depends on $\Delta\Psi$ and ADP availability

$$J_{syn}=k_{syn}\cdot ADP\cdot\Delta\Psi \qquad (41)$$

$k_{syn}=7.5\ mV^{-1}\times s^{-1}$

$$d(C_{ATP})_{ATPsyn}/d(t) = J_{syn}$$
$$d(C_{ADP})_{sATPyn}/d(t) = -J_{syn} \qquad (42)$$
$$d(C_{\Delta\Psi})_{ATPsyn}/d(t) = -8\cdot J_{syn}\cdot F/C$$

The model implementation of electron transport in complexes I, II, and III is described in detail in Text S2.

References

1. Zeylemaker WP, Slater EC (1967) The inhibition of succinate dehydrogenase by oxaloacetate. BBA - Enzymology 132(1):210-212
2. Zagubnaya OA, Boronovskiy SE, Nartsissov YR (2018) Probability analysis of mammalian glutamate transporter activity. Journal of Physics: Conference Series 1141(1)
3. Okubo Y, Sekiya H, Namiki S, Sakamoto H, Iinuma S, Yamasaki M, Watanabe M, Hirose K, Iino M (2010) Imaging extrasynaptic glutamate dynamics in the brain. Proceedings of the National Academy of Sciences of the United States of America 107(14):6526-6531
4. Pinky NF, Wilkie CM, Barnes JR, Parsons MP (2018) Region-and activity-dependent regulation of extracellular glutamate. Journal of Neuroscience 38(23):5351-5366
5. Jones AJY, Blaza JN, Varghese F, Hirst J (2017) Respiratory complex i in bos taurus and paracoccus denitrificans pumps four protons across the membrane for every NADH oxidized. Journal of Biological Chemistry 292(12):4987-4995
6. Cocco T, Lorusso M, Di Paola M, Minuto M, Papa S (1992) Characteristics of energy-linked proton translocation in liposome reconstituted bovine cytochrome bc1 complex: Influence of the protonmotive force on the H+/e− stoichiometry. European Journal of Biochemistry 209(1):475-481
7. Berg J, Liu J, Svahn E, Ferguson-Miller S, Brzezinski P (2020) Structural changes at the surface of cytochrome c oxidase alter the proton-pumping stoichiometry. Biochimica et Biophysica Acta - Bioenergetics 1861(2)
8. Turina P, Samoray D, Gräber P (2003) H+/ATP ratio of proton transport-coupled ATP synthesis and hydrolysis catalysed by CFOF1-liposomes. EMBO Journal 22(3):418-426
9. Guynn RW, Gelberg HJ, Veech RL. Equilibrium constants of the malate dehydrogenase, citrate synthase, citrate lyase, and acetyl coenzyme A hydrolysis reactions under physiological conditions. J Biol Chem. 1973 Oct 25;248(20):6957-65. PMID: 4743509.
